# Supplementary material for: Development and validation of a machine learning model for prediction of type 2 diabetes in patients with mental illness
Source: Acta Psychiatr Scand. 2024 Apr 4;151(3):245–58. doi: 10.1111/acps.13687 (PMC11787919; doi:10.1111/acps.13687)
Supplement: Supplementary file 1 — Data S1. Supporting Information. [file ACPS-151-245-s001.pdf]

Supplemental Online Content

Development and validation of a machine learning model for prediction of type 2 diabetes in patients with mental illness

Martin Bernstorff[1,2,3], Lasse Hansen[1,2,3], Kenneth Enevoldsen[1,2,3], Jakob Damgaard[1,2,3], Frida Hæstrup[1,2,3], Erik Perfalk[1,2], Andreas Aalkjær Danielsen[1,2], Søren Dinesen Østergaard[1,2]

[1] Department of Affective Disorders, Aarhus University Hospital – Psychiatry, Aarhus, Denmark

[2] Department of Clinical Medicine, Aarhus University, Aarhus, Denmark

[3] Center for Humanities Computing, Aarhus University, Denmark

Corresponding author
Martin Bernstorff, MD
Department of Affective Disorders
Aarhus University Hospital - Psychiatry
Palle Juul-Jensens Boulevard 175
8200 Aarhus N
Denmark
E-mail: manber@rm.dk
Telephone: +45 4142 6636

Table of Contents

Supplementary Methods 3

Predictor specification 3

Predictor flattening 3

Descriptive statistics for patients who developed T2D vs. those that did not 3

Hyperparameter tuning 4

Sensitivity analyses 4

Supplementary Figures and Supplementary Tables 5

Supplementary Figure 1. Selection of eligible prediction times before model selection 5

Supplementary Figure 2. First HbA1c ≥ 48 by month for the patients in the dataset 6

Supplementary Figure 3: Cumulative proportion of the last diabetes-measurement within the 5-year lookahead window by classification 7

Supplementary Figure 4: Performance of XGBoost at a 3% predicted positive rate with 1 year of lookahead 8

Supplementary Figure 5: Robustness of XGBoost at a 3% predicted positive rate with 1 years of lookahead 9

Supplementary Figure 6: Performance of XGBoost at a 3% predicted positive rate with 2 years of lookahead 10

Supplementary Figure 7: Robustness of XGBoost at a 3% predicted positive rate with 2 years of lookahead 11

Supplementary Figure 8: Performance of XGBoost at a 3% predicted positive rate with 3 years of lookahead 12

Supplementary Figure 9: Robustness of XGBoost at a 3% predicted positive rate with 3 years of lookahead 13

Supplementary Figure 10: Performance of XGBoost at a 3% predicted positive rate with 4 years of lookahead 14

Supplementary Figure 11: Robustness of XGBoost at a 3% predicted positive rate with 4 years of lookahead 15

Supplementary Figure 12. Performance of XGBoost using only age, sex and HbA1c with 4 years of lookahead 16

Supplementary Figure 13: Robustness of XGBoost using only age, sex and HbA1c with 4 years of lookahead 17

Supplementary Table 1. ICD-10 codes for diabetes......18

Supplementary Table 2: Descriptive statistics for predictors (a list of abbreviations is inserted below the table)......19

Supplementary Table 3. Hyperparameters for model selection. Parameters for the best performing model in bold.......70

Supplementary Table 4. Descriptive statistics for patients that were eligible for prediction, divided by whether they developed T2D or not.......71

Supplementary Table 5. The 100 most important features by information gain (XGBoost with 5 year lookahead)......72

Supplementary Table 6: Performance by predicted positive rate of XGBoost with 1 years of lookahead.......75

Supplementary Table 7: Performance by predicted positive rate of XGBoost with 2 years of lookahead.......76

Supplementary Table 8: Performance by predicted positive rate of XGBoost with 3 years of lookahead.......77

Supplementary Table 9: Performance by predicted positive rate of XGBoost with 4 years of lookahead.......78

Supplementary Table 10: Performance by predicted positive rate of XGBoost with only age, sex and HbA1c with 4 years of lookahead......79

Supplementary Discussion......80

Selection of wash-in periods......80

Validity of the definition of T2D......80

Effects of potential model implementation......80

Supplementary Methods

Predictor specification

Supplementary Table 2 has many predictors which did not have values within the lookbehind window. These do not, however, represent missing data in the traditional sense as they do not result from data that were not entered, but from an actual absence of data. This absence reflects clinical practice and should, therefore, be included, as it matches implementation of a potentially predictive model into clinical practice. However, during pre-processing, imputation may improve performance, and was added.

Predictor flattening

All predictors are time-series. For example, each HbA1c measurement includes both the time it was measured as well as its value. Traditional machine learning models require that each prediction time, e.g., each psychiatric service contact, be represented as one row. However, each HbA1c measurement might be relevant to more than one prediction time. Furthermore, more than one measurement might be relevant to the same prediction time. This means we need a many-to-many join, filter by time, and aggregate within this time filter. Specifically, we looked back a given distance from the prediction time (lookbehind window), and aggregated the measurements within this window (Figure 1F) using the "timeseriesflattener" Python package.[1] The final dataset was quality checked manually by inspecting features, as well as with the Deepchecks suite v0.13.1. It passed all relevant checks, e.g. lack of patient overlap between splits and no predictor being too strongly correlated with the outcome as a sign of leakage.[2]

Descriptive statistics for patients who developed T2D vs. those that did not

Supplementary Table 4 lists the characteristics of patients developing T2D compared to those who did not. The patients who developed T2D differed from those who did not by being more likely to be male and being older, two well-known risk factors for T2D.[3] With respect to psychiatric diagnoses, patients who developed T2D were more likely to be diagnosed with organic mental disorders (F0), which are prevalent among the elderly. Patients who developed T2D also had a slightly lower proportion of neurotic, stress-related and somatoform disorders (F4), disorders of adult personality and behaviour (F6) and

developmental disorders (F8), which is likely explained by the lower average age of patients within these disorders. With respect to psychopharmacological treatment, patients who developed T2D were also more likely to receive any of the top 10 weight gaining antipsychotics, which are likely to increase the risk of T2D.[4] Moreover, patients who developed T2D were more likely to receive psychopharmacological treatment of any kind, indicating that they had higher psychiatric morbidity, which is linked to risk factors for T2D.[5]

Hyperparameter tuning

For each lookahead window, the algorithm optimised settings for lookbehind combinations, predictor selection methods, imputation methods, and model hyperparameters (see Supplementary Table 2 for a full description of all settings used for experiments). The search was conducted for 12+ hours on a server with a 30-core Intel Xeon CPU, 512GB of RAM and an NVIDIA A100 GPU, resulting in approximately 250 trained models per lookahead window. Training was terminated when the increase in AUROC over the last 2 hours of training was <0.001. Models were trained using 5-fold cross-validation. The code for the full project is available on Github: https://github.com/Aarhus-Psychiatry-Research/psycop-common/tree/main/src/psycop/projects/t2d

Sensitivity analyses

The following sensitivity analyses were carried out: First, as it could be expected that the T2D pattern would vary across age, sex, and number of prior HbA1c-measurements, we examined predictive performance across these characteristics. Second, we examined the temporal stability of predictions by plotting AUROC across time since first contact to the Psychiatric Services of the Central Denmark Region, as well as examined periodicity across months of the year and days of the week. Third, model performance with only sex, age, and HbA1c as predictors was assessed to estimate the additional value of adding a rich feature set. Fourth, since prediction models may issue predictions based on clinical suspicion, we reran the analysis only providing the model with information on whether a measurement was carried out or not – without providing the value/result of the measurement. This imitates the information about the clinical suspicion that the model is provided with. For instance, if the healthcare staff frequently measures the patient's LDL-level, it likely reflects suspicion of unhealthy lifestyle or predisposition, irrespective of whether LDL is elevated.

Supplementary Figures and Supplementary Tables

Supplementary Figure 1. Selection of eligible prediction times before model selection

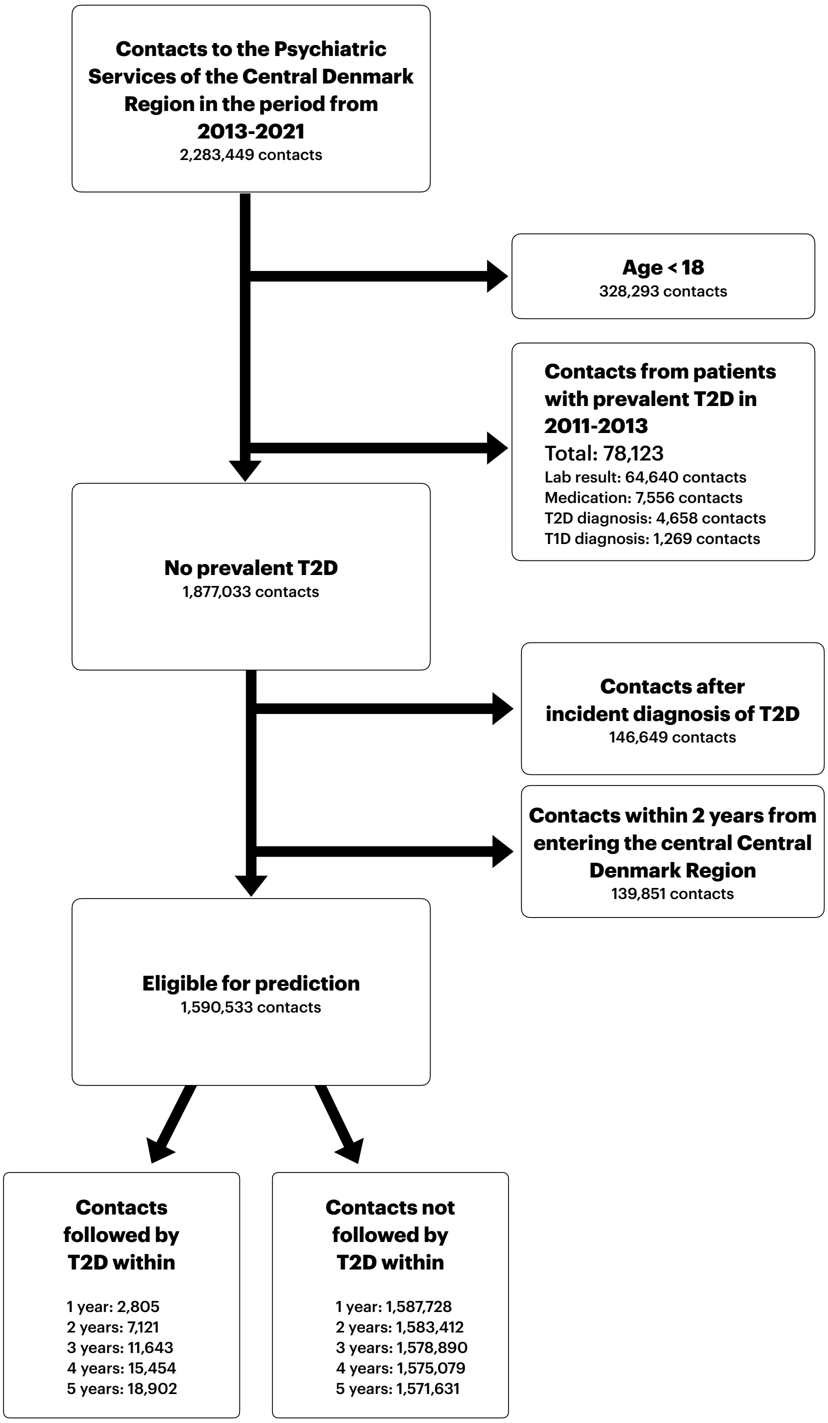

Note that prediction time filtering based on sufficient lookbehind and lookahead (see Figure 1B) happens after the selection illustrated by this figure. As such, counts will differ based on pipeline lookahead and lookbehind. For example, since a total of 8 years of data is available, a model with 2 years of lookbehind and 5 years of lookahead will filter out (5+2)/8 = 87.5% of the eligible prediction times due to insufficient data.

Supplementary Figure 2. First HbA1c ≥ 48 by month for the patients in the dataset

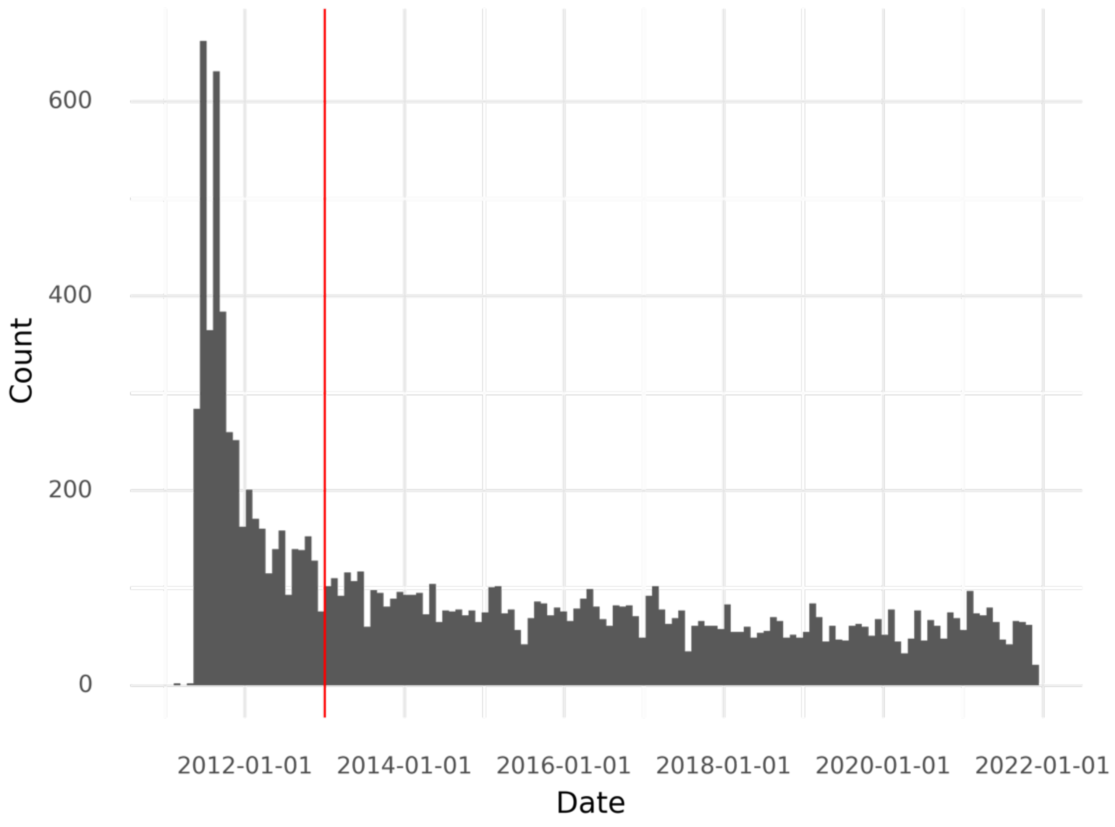

The red line represents the end of the wash-in period (January 1st 2013).

Supplementary Figure 3: Cumulative proportion of the last diabetes-measurement within the 5-year lookahead window by classification

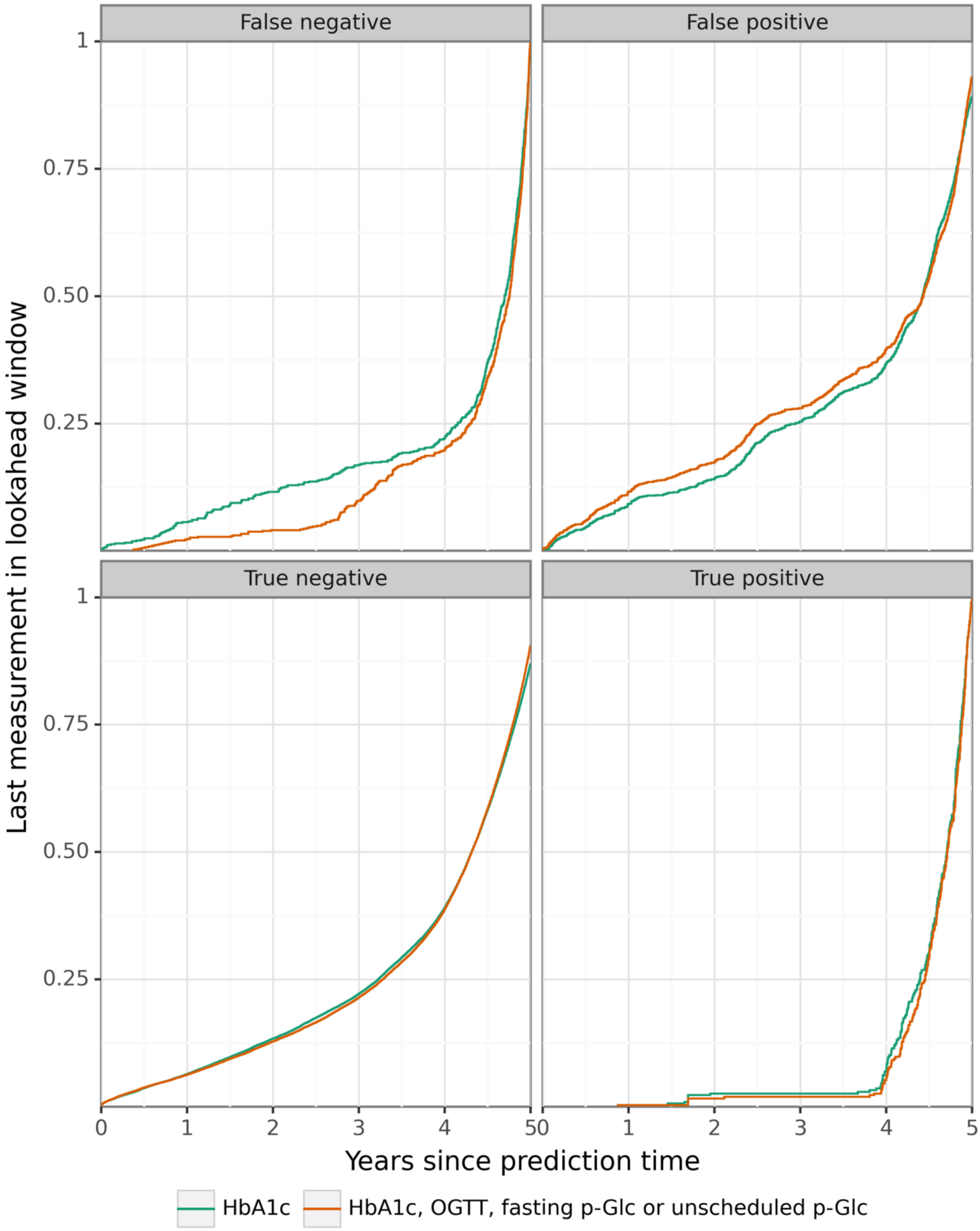

HbA1c: Haemoglobin A1c, **OGTT:** Oral glucose tolerance test, **p-Glc:** Plasma glucose.

Supplementary Figure 4: Performance of XGBoost at a 3% predicted positive rate with 1 year of lookahead

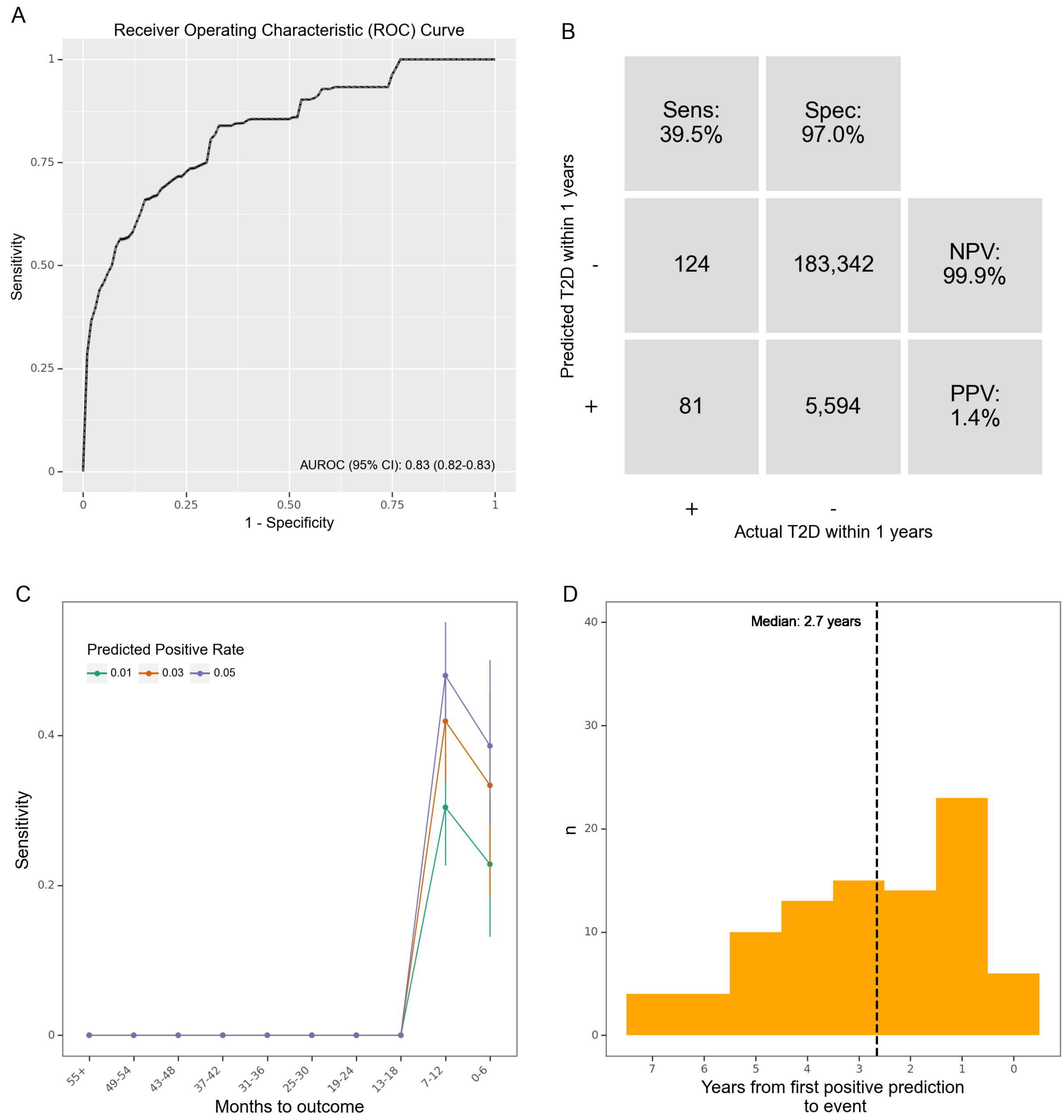

A: Receiver operating characteristics (ROC) curve. **B**: Confusion matrix. PPV: Positive predictive value. NPV: Negative predictive value. **C**: Sensitivity by months from prediction time to event, stratified by desired predicted positive rate (PPR). Note that the numbers do not match those in Table 1, since all prediction times with insufficient lookahead distance have been dropped. **D**: Time (years) from the first positive prediction to the patient having developed T2D at a 3% predicted positive rate (PPR).

Supplementary Figure 5: Robustness of XGBoost at a 3% predicted positive rate with 1 years of lookahead

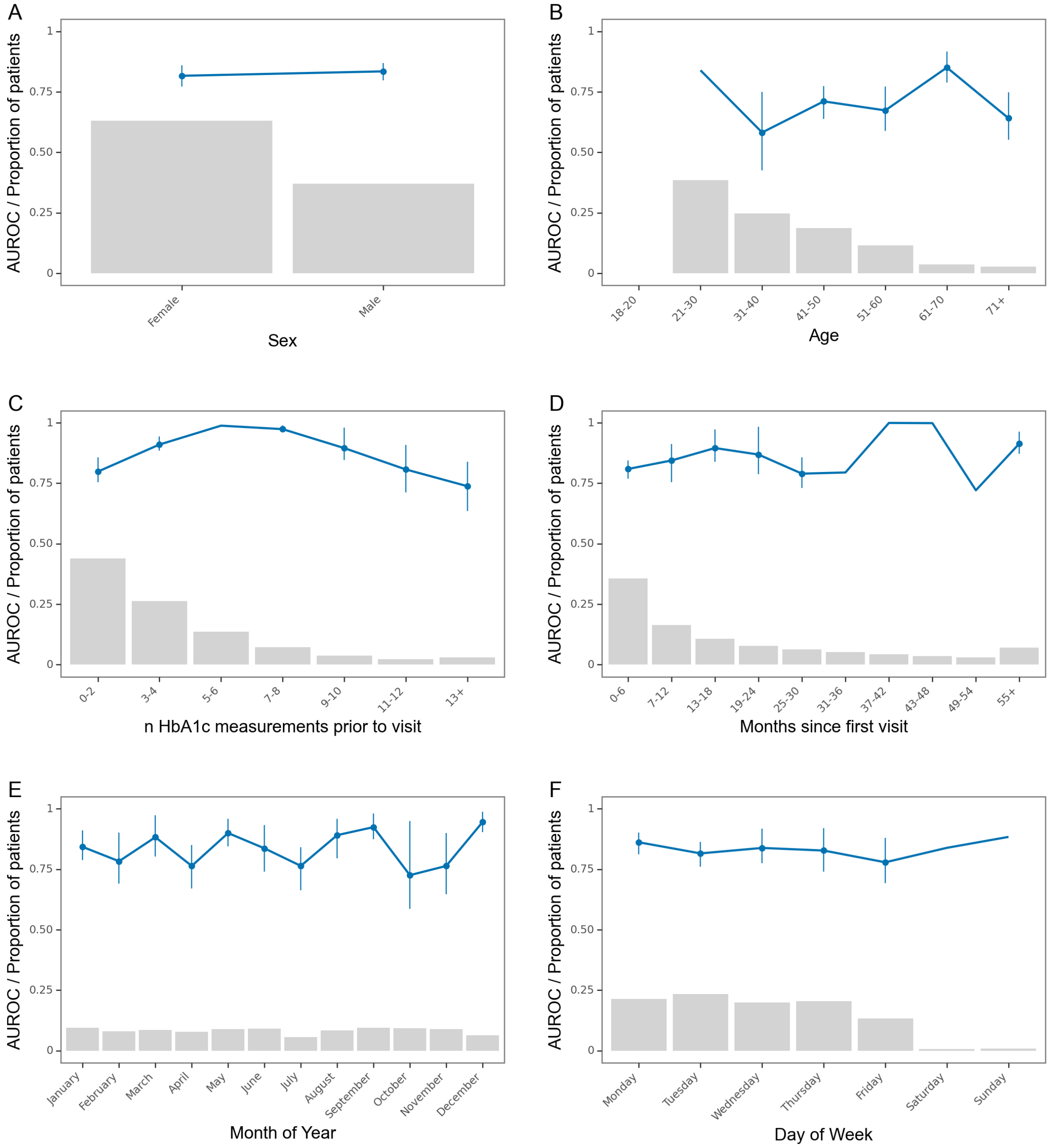

Robustness of the model stratifications. Blue line is the area under the receiver operating characteristics curve. Grey bars represent the proportion of visits that are present in each group. Error bars are 95%-confidence intervals from 100-fold bootstrap.

Supplementary Figure 6: Performance of XGBoost at a 3% predicted positive rate with 2 years of lookahead

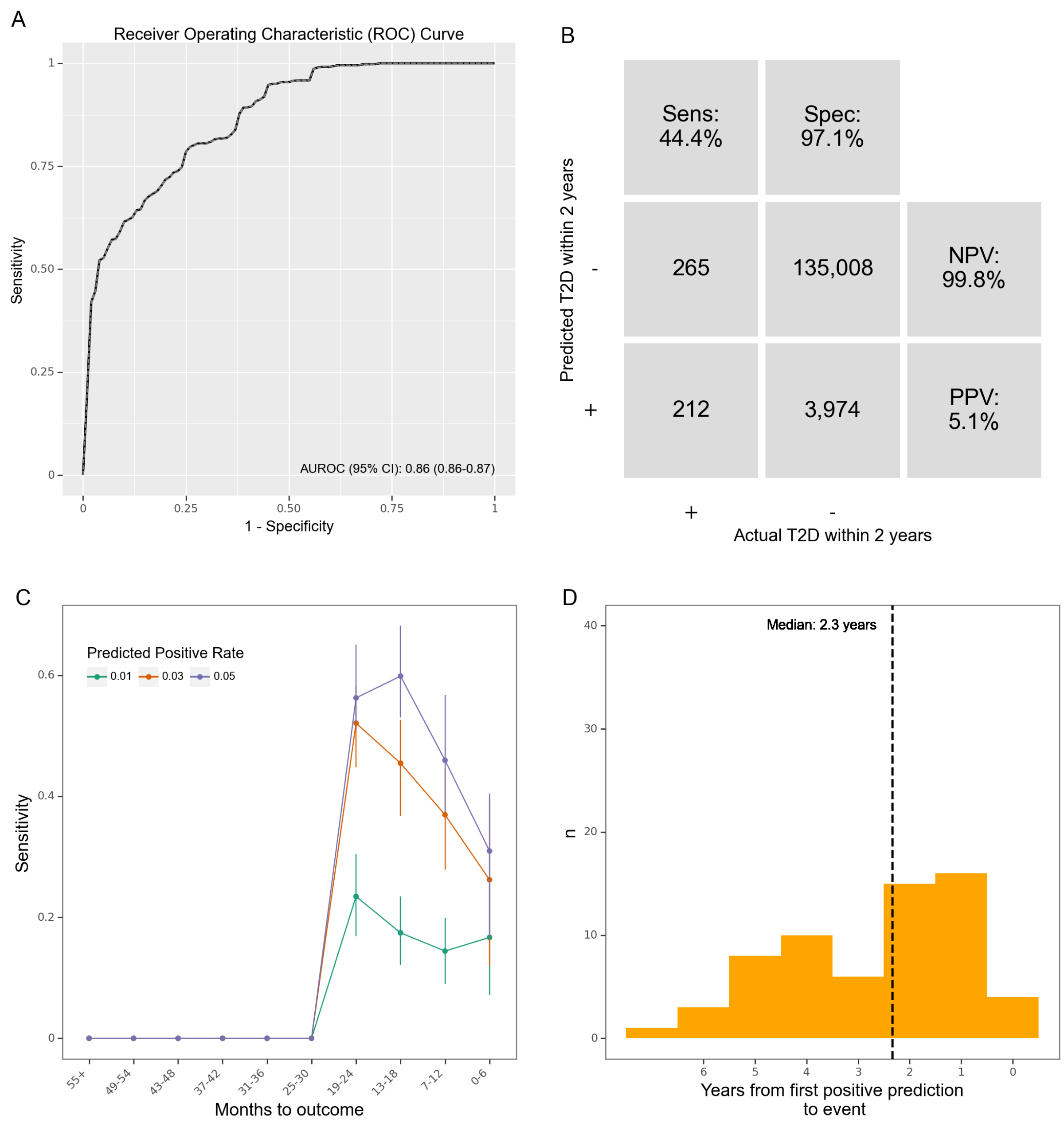

A: Receiver operating characteristics (ROC) curve. **B**: Confusion matrix. PPV: Positive predictive value. NPV: Negative predictive value. **C**: Sensitivity by months from prediction time to event, stratified by desired predicted positive rate (PPR). Note that the numbers do not match those in Table 1, since all prediction times with insufficient lookahead distance have been dropped. **D**: Time (years) from the first positive prediction to the patient having developed T2D at a 3% predicted positive rate (PPR).

Supplementary Figure 7: Robustness of XGBoost at a 3% predicted positive rate with 2 years of lookahead

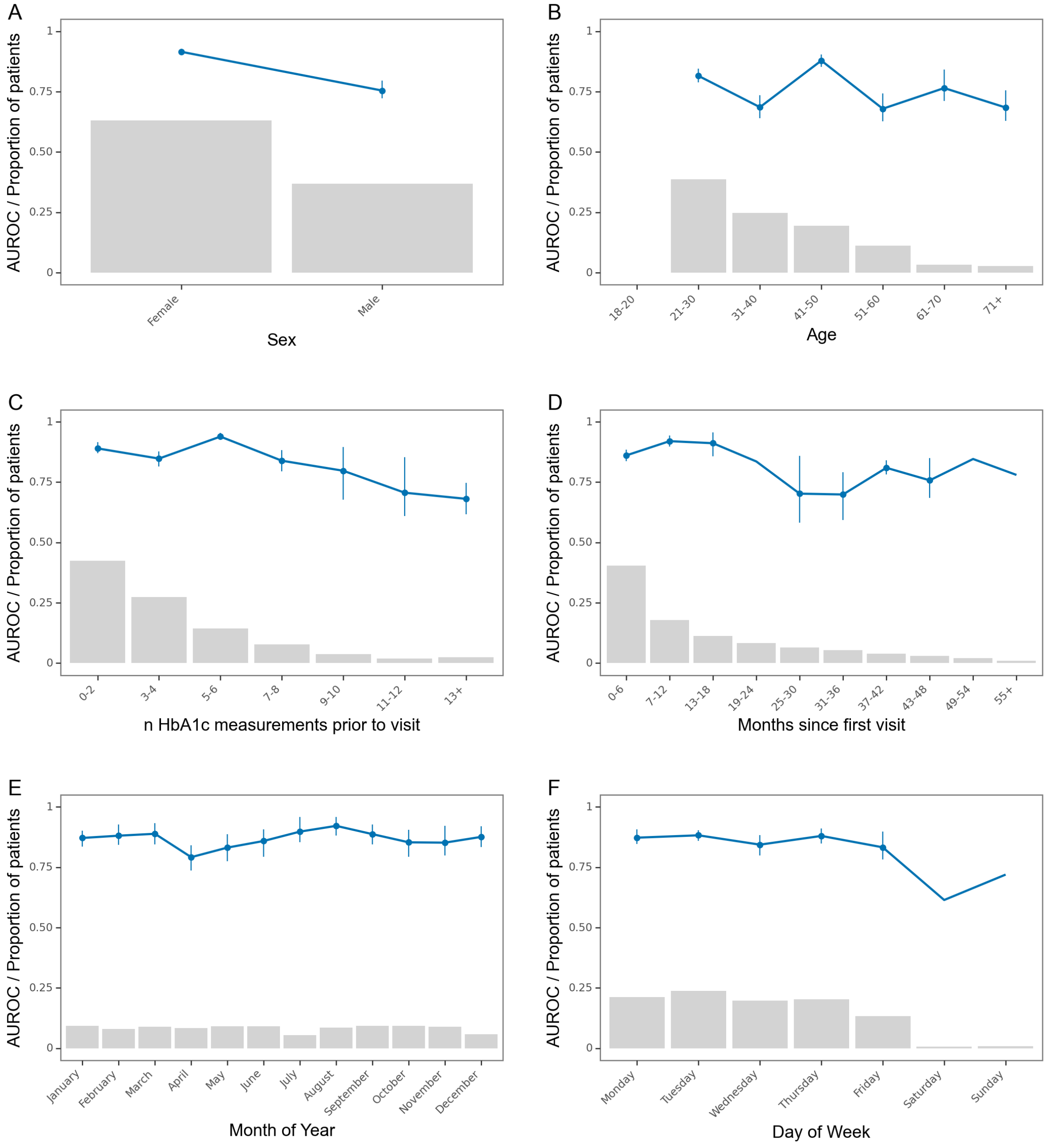

Robustness of the model stratifications. Blue line is the area under the receiver operating characteristics curve. Grey bars represent the proportion of visits that are present in each group. Error bars are 95%-confidence intervals from 100-fold bootstrap.

Supplementary Figure 8: Performance of XGBoost at a 3% predicted positive rate with 3 years of lookahead

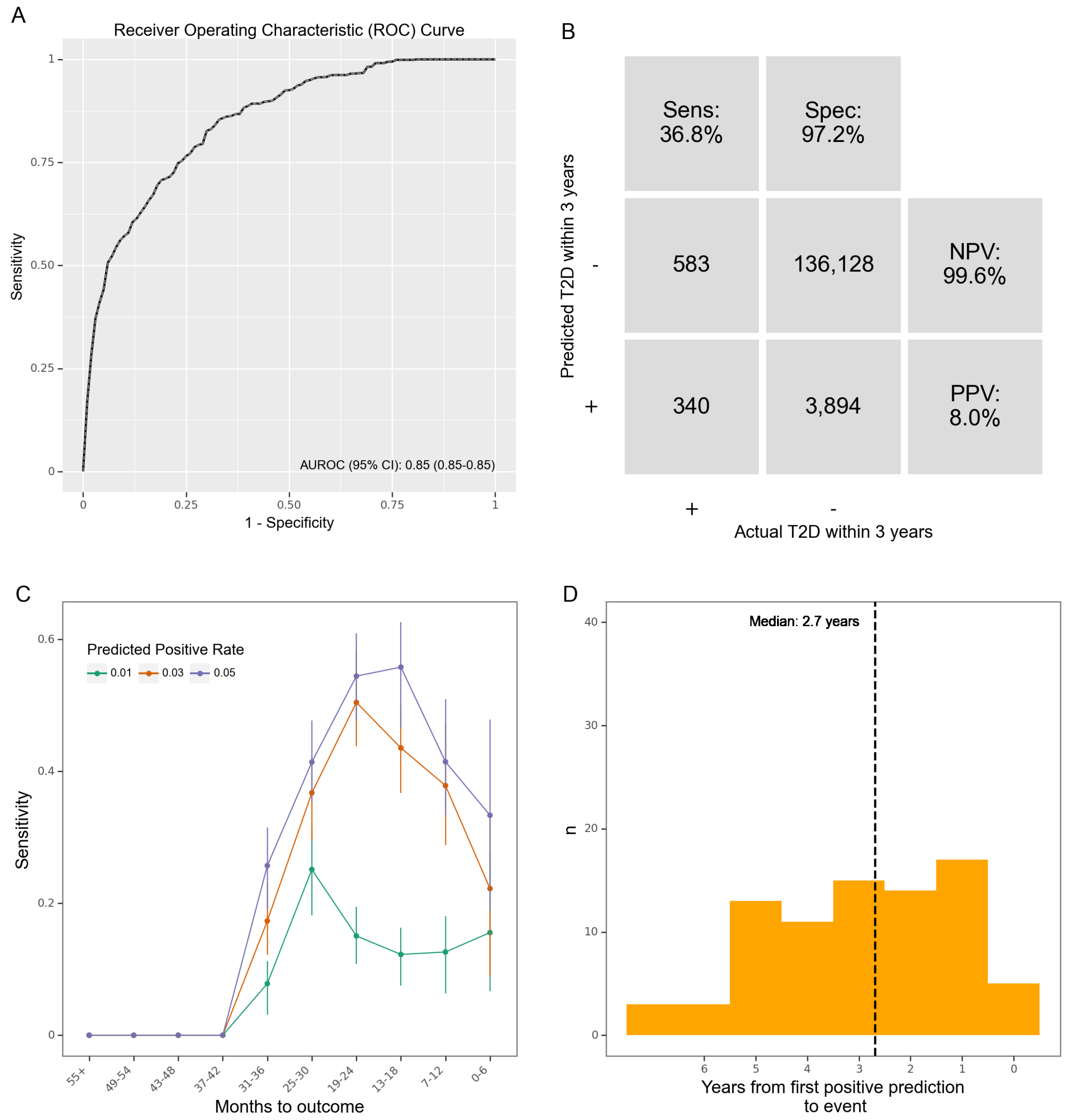

A: Receiver operating characteristics (ROC) curve. **B**: Confusion matrix. PPV: Positive predictive value. NPV: Negative predictive value. **C**: Sensitivity by months from prediction time to event, stratified by desired predicted positive rate (PPR). Note that the numbers do not match those in Table 1, since all prediction times with insufficient lookahead distance have been dropped. **D**: Time (years) from the first positive prediction to the patient having developed T2D at a 3% predicted positive rate (PPR).

Supplementary Figure 9: Robustness of XGBoost at a 3% predicted positive rate with 3 years of lookahead

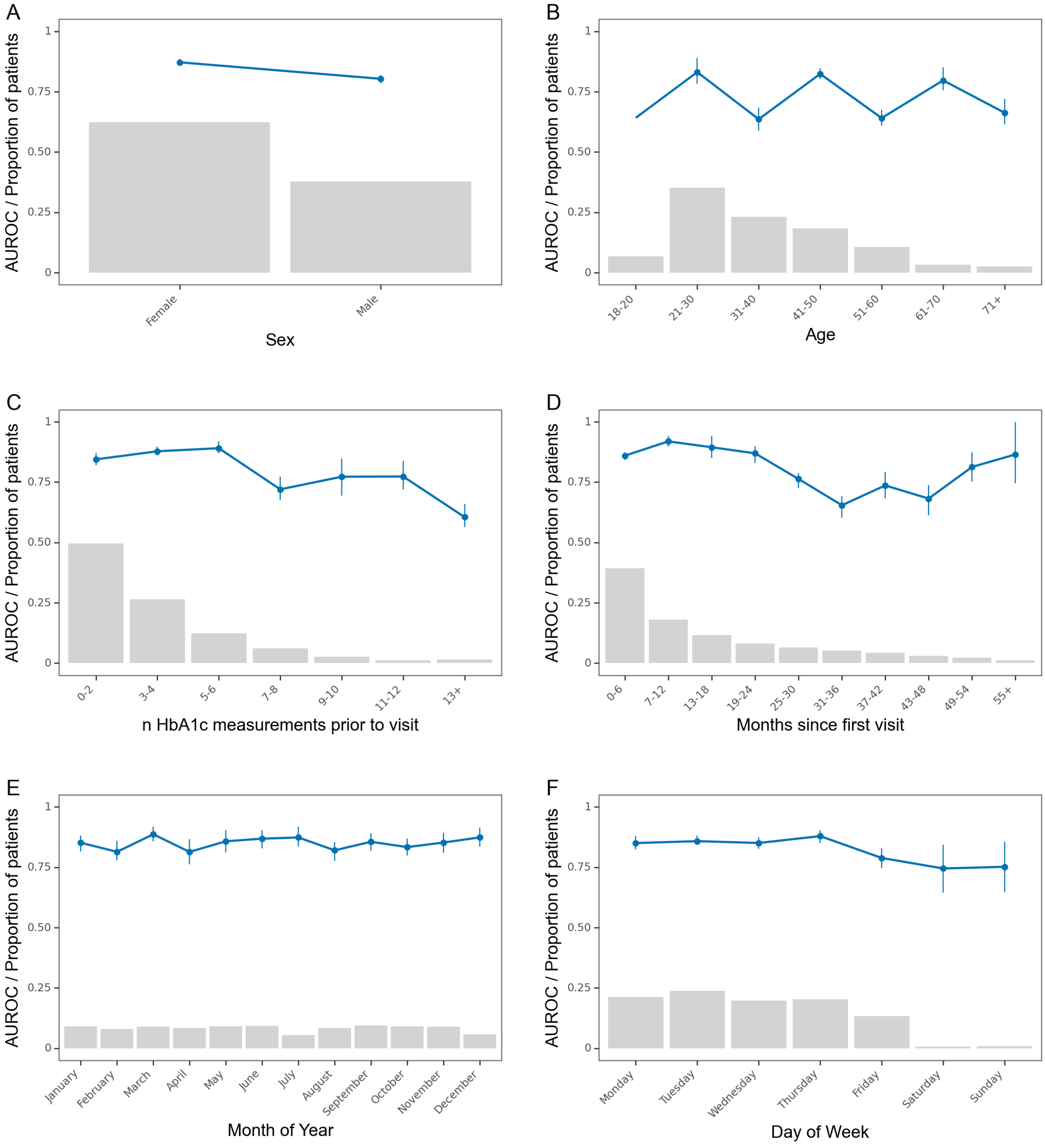

Robustness of the model stratifications. Blue line is the area under the receiver operating characteristics curve. Grey bars represent the proportion of visits that are present in each group. Error bars are 95%-confidence intervals from 100-fold bootstrap.

Supplementary Figure 10: Performance of XGBoost at a 3% predicted positive rate with 4 years of lookahead

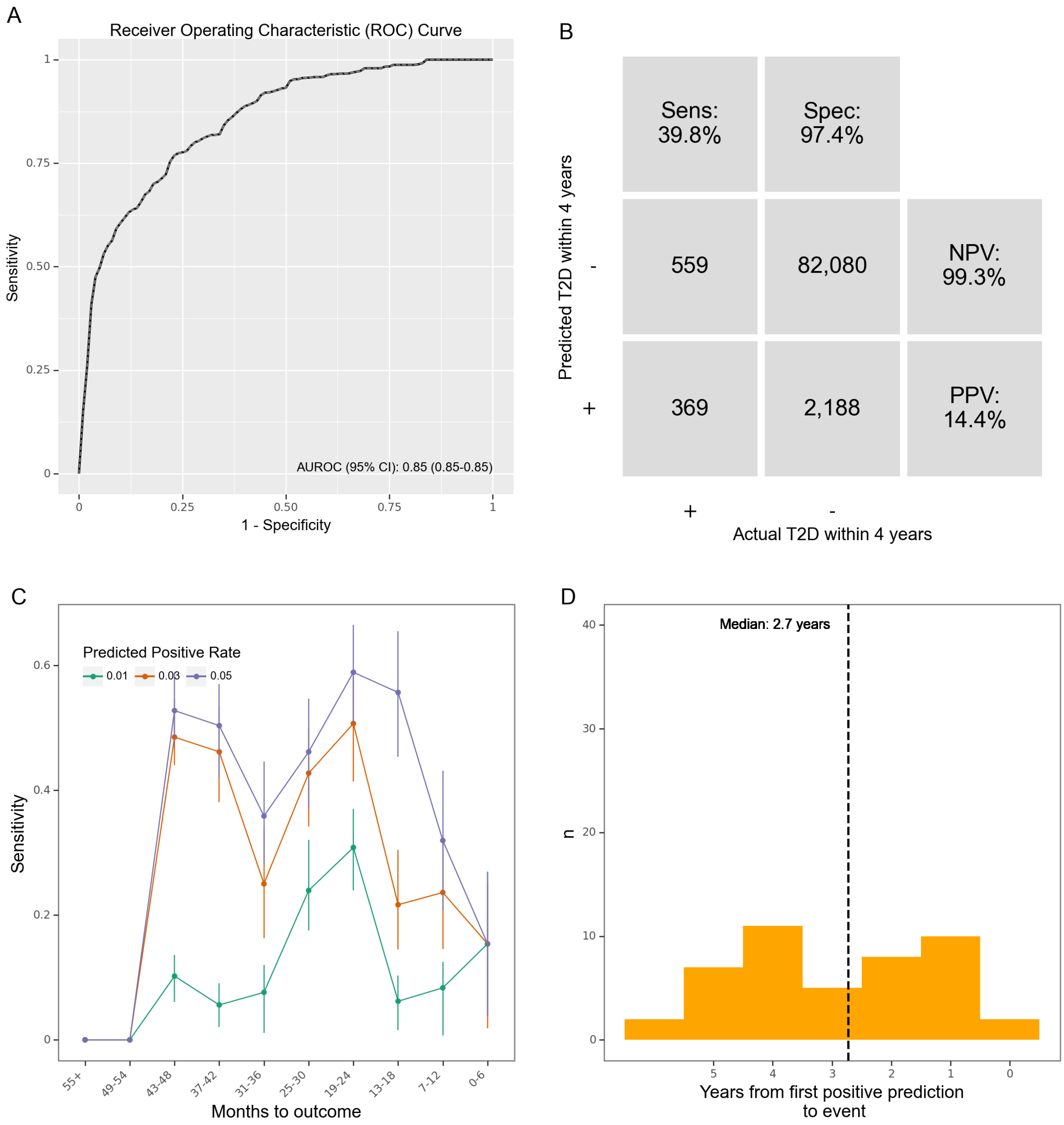

A: Receiver operating characteristics (ROC) curve. **B**: Confusion matrix. PPV: Positive predictive value. NPV: Negative predictive value. **C**: Sensitivity by months from prediction time to event, stratified by desired predicted positive rate (PPR). Note that the numbers do not match those in Table 1, since all prediction times with insufficient lookahead distance have been dropped. **D**: Time (years) from the first positive prediction to the patient having developed T2D at a 3% predicted positive rate (PPR).

Supplementary Figure 11: Robustness of XGBoost at a 3% predicted positive rate with 4 years of lookahead.

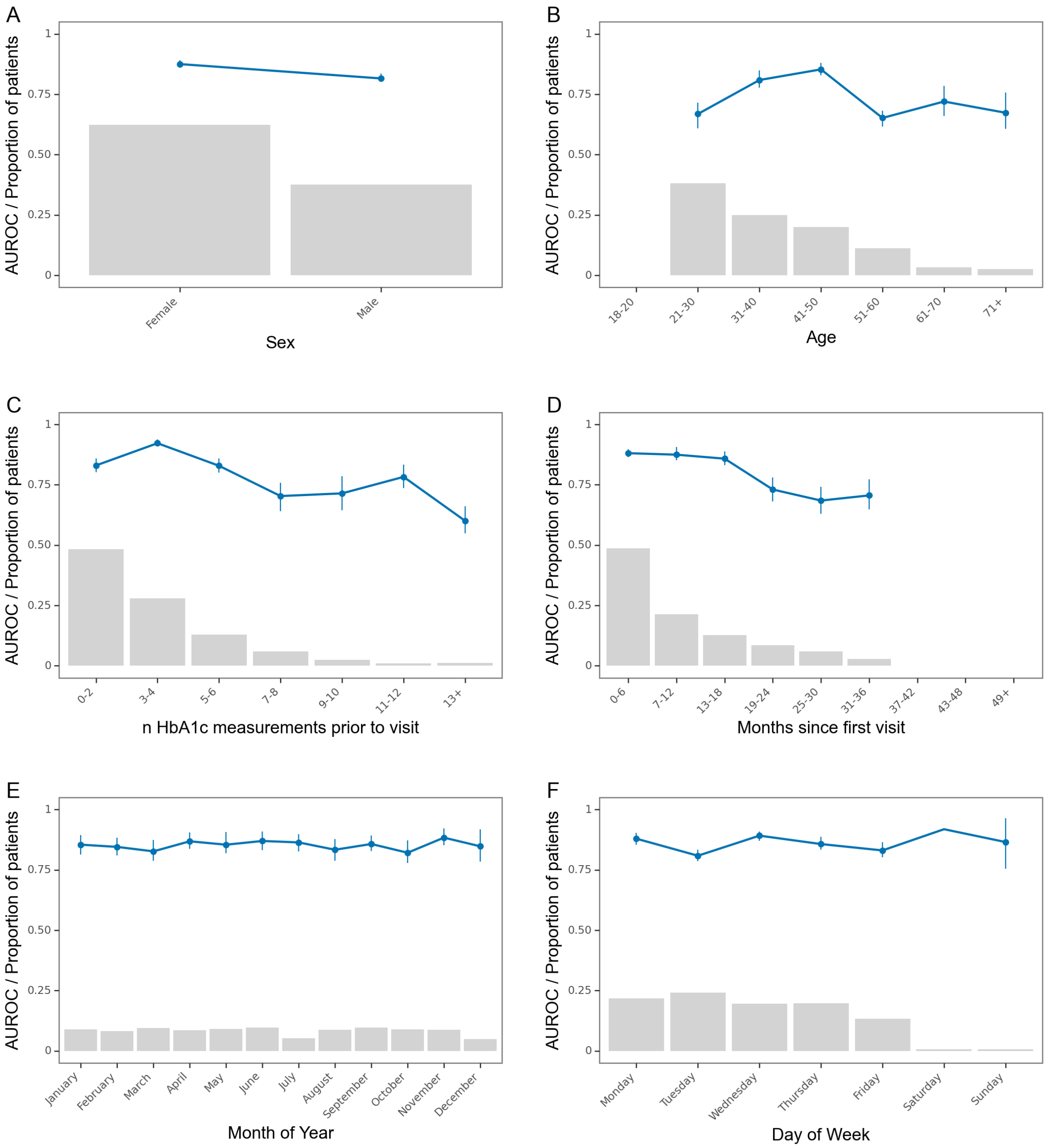

Robustness of the model stratifications. Blue line is the area under the receiver operating characteristics curve. Grey bars represent the proportion of visits that are present in each group. Error bars are 95%-confidence intervals from 100-fold bootstrap

Supplementary Figure 12. Performance of XGBoost using only age, sex and HbA1c with 4 years of lookahead

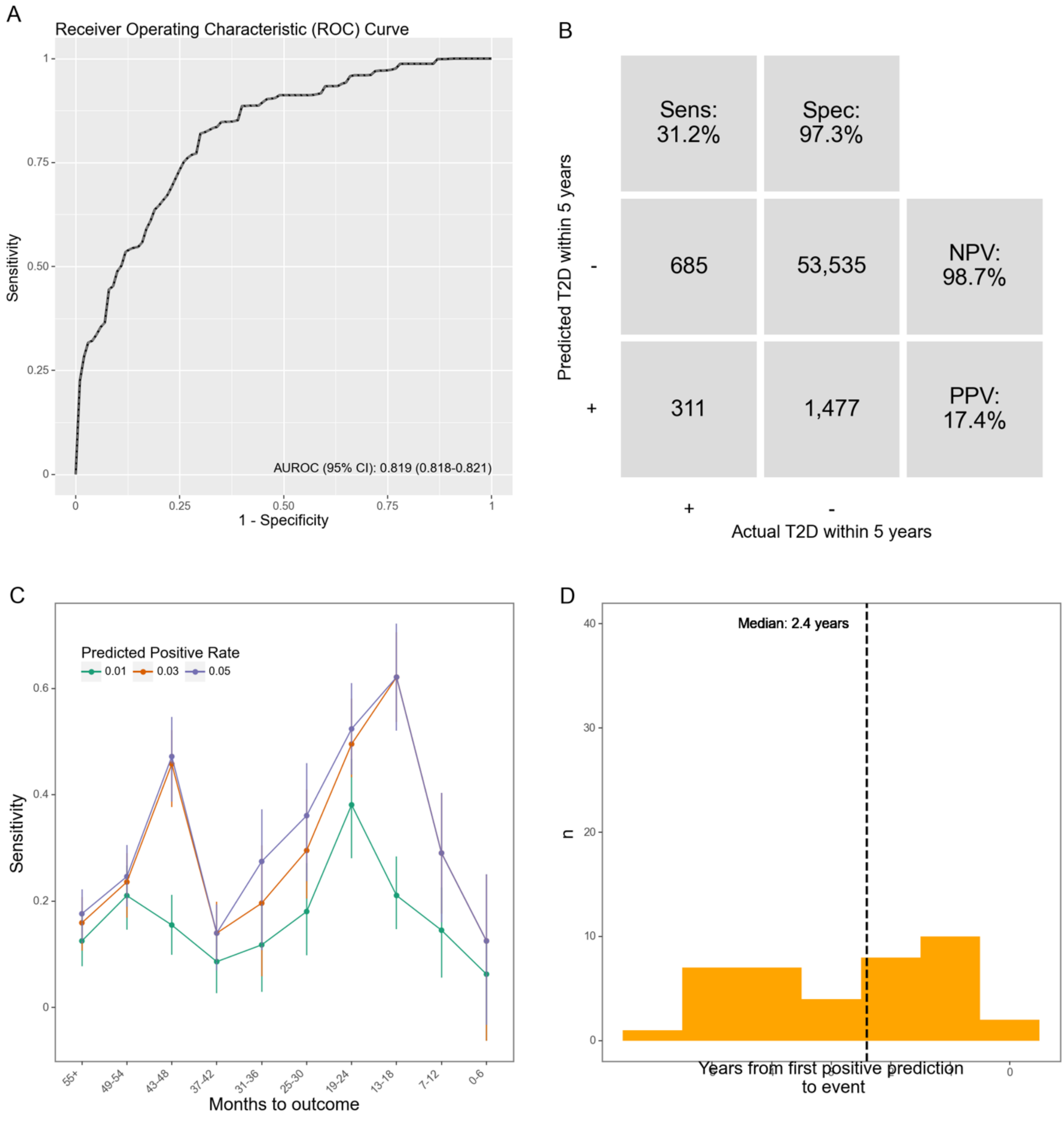

A: Receiver operating characteristics (ROC) curve. **B**: Confusion matrix. PPV: Positive predictive value. NPV: Negative predictive value. **C**: Sensitivity by months from prediction time to event, stratified by desired predicted positive rate (PPR). Note that the numbers do not match those in Table 1, since all prediction times with insufficient lookahead distance have been dropped. **D**: Time (years) from the first positive prediction to the patient having developed T2D at a 3% predicted positive rate (PPR).

Supplementary Figure 13: Robustness of XGBoost using only age, sex and HbA1c with 4 years of lookahead

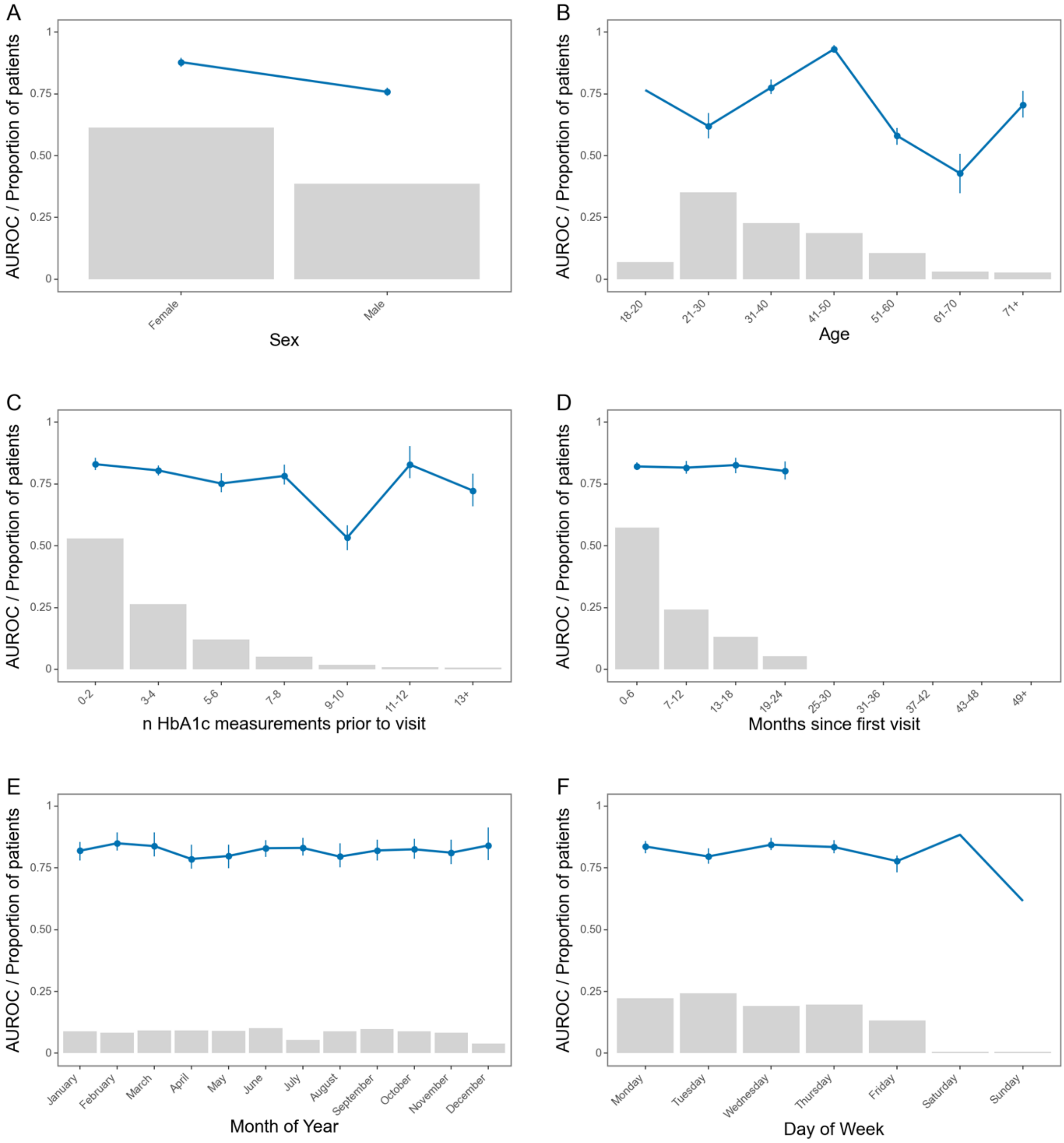

Robustness of the model stratifications. Blue line is the area under the receiver operating characteristics curve. Grey bars represent the proportion of visits that are present in each group. Error bars are 95%-confidence intervals from 100-fold bootstrap

Supplementary Table 1. ICD-10 codes for diabetes

E11.*, E12.*, E13.*, E14.*, E15.*	Diabetes
E16.0-E16.2	Diabetic mononeuropathy
G63.2	Diabetic polyneuropathy
H28.0	Diabetic cataract
H33.4B	Diabetic retinal amotio
H36.0	Diabetic retinopathy
M14.2	Diabetic arthropathy
N08.3	Diabetic glomerulonephropathy
O24.*	Diabetes mellitus in pregnancy
T38.3A	Insulin shock

Supplementary Table 2: Descriptive statistics for predictors (a list of abbreviations is inserted below the table)

Supplementary Table 2: Descriptive statistics for predictors (a list of abbreviations is inserted below the table)

Predictor	Lookbehind days	Aggregation function	Unique values in predictor	Fallback strategy	Mean	Proportion using fallback	25-percentile	50-percentile	75-percentile
alat	30	maximum	510	nan	32.29	0.82	15	21	32
alat	30	minimum	379	nan	27.17	0.82	14	20	31
alat	1095	minimum	231	nan	18.73	0.07	11	15	22
alat	730	minimum	253	nan	19.93	0.1	12	16	23
alat	365	minimum	290	nan	22.06	0.21	13	18	26
alat	180	minimum	327	nan	23.84	0.39	14	19	28
alat	1095	maximum	765	nan	48.7	0.07	19	27	44
alat	1825	maximum	792	nan	54.01	0.06	20	29	48
alat	730	maximum	735	nan	44.42	0.1	18	26	41
alat	365	maximum	681	nan	38.55	0.21	16	23	37
alat	180	maximum	614	nan	35.18	0.39	16	22	34
alat	1825	minimum	218	nan	17.6	0.06	11	15	21
alat	1460	minimum	223	nan	18.04	0.06	11	15	21
alat	1825	mean	24744	nan	28.96	0.06	15.5	21.2	31
alat	1460	maximum	780	nan	51.66	0.06	19	28	47
alat	180	mean	6445	nan	28.2	0.39	15	21	31
alat	30	mean	1940	nan	29.42	0.82	15	21	31.5
alat	1825	latest	467	nan	25.96	0.06	14	20	30
alat	1460	latest	467	nan	25.97	0.06	14	20	30
alat	1095	latest	467	nan	26	0.07	14	20	30
alat	365	latest	464	nan	26.26	0.21	14	20	30
alat	180	latest	459	nan	26.6	0.39	15	20	31
alat	730	latest	465	nan	26.07	0.1	14	20	30

alat	1460	mean	23198	nan	28.85	0.06	15.5	21.1	31
alat	1095	mean	20409	nan	28.69	0.07	15.3	21	31
alat	730	mean	16304	nan	28.51	0.1	15	21	31
alat	365	mean	10417	nan	28.19	0.21	15	21	31
alat	30	latest	439	nan	28.26	0.82	15	21	31
albumine_creatinine_ratio	180	maximum	410	nan	77.18	0.98	3.4	7	14
albumine_creatinine_ratio	1460	maximum	493	nan	58.96	0.93	4	7	15
albumine_creatinine_ratio	365	maximum	439	nan	64.57	0.97	3.4	6.7	14
albumine_creatinine_ratio	730	maximum	464	nan	60.29	0.95	4	7	14
albumine_creatinine_ratio	1825	minimum	331	nan	18.67	0.93	3	5	9.4
albumine_creatinine_ratio	1095	maximum	481	nan	57.99	0.94	4	7	14.1
albumine_creatinine_ratio	1825	maximum	497	nan	58.02	0.93	4	7	15.4
albumine_creatinine_ratio	180	minimum	375	nan	46.1	0.98	3.4	6.7	13
albumine_creatinine_ratio	365	minimum	382	nan	30.51	0.97	3	6	12
albumine_creatinine_ratio	730	minimum	366	nan	24.25	0.95	3	5	11
albumine_creatinine_ratio	1095	minimum	343	nan	20.5	0.94	3	5	10
albumine_creatinine_ratio	30	maximum	296	nan	93.35	1	4	7	16
albumine_creatinine_ratio	30	minimum	291	nan	87.91	1	4	7	16
albumine_creatinine_ratio	1460	minimum	339	nan	19.56	0.93	3	5	10
albumine_creatinine_ratio	1825	latest	498	nan	31.45	0.93	3	6	12
albumine_creatinine_ratio	180	latest	414	nan	56.63	0.98	3.4	6.7	14
albumine_creatinine_ratio	30	mean	315	nan	90.72	1	4	7	16
albumine_creatinine_ratio	1825	mean	2100	nan	33.32	0.93	3.5	6	13
albumine_creatinine_ratio	730	mean	1404	nan	38.51	0.95	3.4	6	12.7
albumine_creatinine_ratio	1095	mean	1752	nan	35.2	0.94	3.4	6	12.5
albumine_creatinine_ratio	1460	mean	1945	nan	34.57	0.93	3.5	6	13
albumine_creatinine_ratio	30	latest	294	nan	88.13	1	4	7	16
albumine_creatinine_ratio	365	mean	971	nan	45.09	0.97	3.4	6	13

albumine_creatinine_ratio	365	latest	459	nan	42.56	0.97	3.4	6	12
albumine_creatinine_ratio	730	latest	478	nan	36.07	0.95	3	6	12
albumine_creatinine_ratio	1095	latest	489	nan	33.44	0.94	3	6	12
albumine_creatinine_ratio	1460	latest	497	nan	32.5	0.93	3	6	12
albumine_creatinine_ratio	180	mean	707	nan	59.86	0.98	3.4	6.7	14
antihypertensives	180	mean	2	0	0	1	0	0	0
antihypertensives	30	mean	2	0	0	1	0	0	0
antihypertensives	1460	minimum	2	0	0.01	0.99	0	0	0
antihypertensives	1095	minimum	2	0	0.01	0.99	0	0	0
antihypertensives	365	minimum	2	0	0	1	0	0	0
antihypertensives	1460	mean	2	0	0.01	0.99	0	0	0
antihypertensives	30	minimum	2	0	0	1	0	0	0
antihypertensives	365	mean	2	0	0	1	0	0	0
antihypertensives	30	maximum	2	0	0	1	0	0	0
antihypertensives	1460	maximum	2	0	0.01	0.99	0	0	0
antihypertensives	1825	maximum	2	0	0.01	0.99	0	0	0
antihypertensives	180	minimum	2	0	0	1	0	0	0
antihypertensives	730	mean	2	0	0.01	0.99	0	0	0
antihypertensives	730	minimum	2	0	0.01	0.99	0	0	0
antihypertensives	1825	minimum	2	0	0.01	0.99	0	0	0
antihypertensives	365	latest	2	0	0	1	0	0	0
antihypertensives	730	latest	2	0	0.01	0.99	0	0	0
antihypertensives	1095	latest	2	0	0.01	0.99	0	0	0
antihypertensives	180	maximum	2	0	0	1	0	0	0
antihypertensives	1825	latest	2	0	0.01	0.99	0	0	0
antihypertensives	1095	maximum	2	0	0.01	0.99	0	0	0
antihypertensives	180	latest	2	0	0	1	0	0	0
antihypertensives	730	maximum	2	0	0.01	0.99	0	0	0

antihypertensives	365	maximum	2	0	0	1	0	0	0
antihypertensives	30	latest	2	0	0	1	0	0	0
antihypertensives	1460	latest	2	0	0.01	0.99	0	0	0
antihypertensives	1825	mean	2	0	0.01	0.99	0	0	0
antihypertensives	1095	mean	2	0	0.01	0.99	0	0	0
antipsychotics	1095	maximum	2	0	0.3	0.7	0	0	1
antipsychotics	730	maximum	2	0	0.27	0.73	0	0	1
antipsychotics	365	maximum	2	0	0.22	0.78	0	0	0
antipsychotics	180	maximum	2	0	0.17	0.83	0	0	0
antipsychotics	30	maximum	2	0	0.08	0.92	0	0	0
antipsychotics	1825	minimum	2	0	0.32	0.68	0	0	1
antipsychotics	1460	latest	2	0	0.31	0.69	0	0	1
antipsychotics	1095	latest	2	0	0.3	0.7	0	0	1
antipsychotics	730	latest	2	0	0.27	0.73	0	0	1
antipsychotics	365	latest	2	0	0.22	0.78	0	0	0
antipsychotics	1460	maximum	2	0	0.31	0.69	0	0	1
antipsychotics	30	latest	2	0	0.08	0.92	0	0	0
antipsychotics	1825	maximum	2	0	0.32	0.68	0	0	1
antipsychotics	180	latest	2	0	0.17	0.83	0	0	0
antipsychotics	180	minimum	2	0	0.17	0.83	0	0	0
antipsychotics	1825	latest	2	0	0.32	0.68	0	0	1
antipsychotics	1825	mean	2	0	0.32	0.68	0	0	1
antipsychotics	1460	mean	2	0	0.31	0.69	0	0	1
antipsychotics	1095	mean	2	0	0.3	0.7	0	0	1
antipsychotics	730	mean	2	0	0.27	0.73	0	0	1
antipsychotics	365	mean	2	0	0.22	0.78	0	0	0
antipsychotics	30	minimum	2	0	0.08	0.92	0	0	0
antipsychotics	30	mean	2	0	0.08	0.92	0	0	0

antipsychotics	1460	minimum	2	0	0.31	0.69	0	0	1
antipsychotics	1095	minimum	2	0	0.3	0.7	0	0	1
antipsychotics	730	minimum	2	0	0.27	0.73	0	0	1
antipsychotics	365	minimum	2	0	0.22	0.78	0	0	0
antipsychotics	180	mean	2	0	0.17	0.83	0	0	0
arterial_p_glc	1460	minimum	119	nan	5.88	0.9	5.1	5.6	6.4
arterial_p_glc	1095	minimum	116	nan	5.91	0.91	5.1	5.6	6.5
arterial_p_glc	730	minimum	118	nan	5.94	0.93	5.1	5.7	6.5
arterial_p_glc	365	minimum	114	nan	5.97	0.95	5.2	5.7	6.6
arterial_p_glc	30	maximum	136	nan	6.8	0.99	5.5	6.3	7.5
arterial_p_glc	180	maximum	162	nan	7.02	0.97	5.6	6.5	7.8
arterial_p_glc	180	minimum	111	nan	5.98	0.97	5.2	5.7	6.6
arterial_p_glc	730	maximum	178	nan	7.19	0.93	5.7	6.7	8.1
arterial_p_glc	1460	maximum	179	nan	7.26	0.9	5.8	6.7	8.2
arterial_p_glc	730	mean	1625	nan	6.47	0.93	5.6	6.2	7.2
arterial_p_glc	1095	maximum	179	nan	7.24	0.91	5.7	6.7	8.1
arterial_p_glc	1825	minimum	120	nan	5.87	0.89	5.1	5.6	6.4
arterial_p_glc	365	maximum	172	nan	7.12	0.95	5.7	6.6	7.9
arterial_p_glc	365	mean	1340	nan	6.46	0.95	5.5	6.2	7.2
arterial_p_glc	30	mean	664	nan	6.36	0.99	5.4	6.1	7
arterial_p_glc	1095	mean	1772	nan	6.47	0.91	5.6	6.2	7.1
arterial_p_glc	180	mean	1068	nan	6.43	0.97	5.5	6.2	7.1
arterial_p_glc	1825	maximum	179	nan	7.28	0.89	5.8	6.7	8.2
arterial_p_glc	1825	latest	128	nan	6.43	0.89	5.4	6.1	7.1
arterial_p_glc	1460	latest	128	nan	6.42	0.9	5.4	6.1	7.1
arterial_p_glc	730	latest	127	nan	6.42	0.93	5.4	6.1	7.1
arterial_p_glc	1095	latest	127	nan	6.43	0.91	5.4	6.1	7.1
arterial_p_glc	180	latest	118	nan	6.38	0.97	5.4	6.1	7

arterial_p_glc	30	latest	108	nan	6.31	0.99	5.4	6	6.9
arterial_p_glc	1825	mean	1914	nan	6.47	0.89	5.6	6.2	7.1
arterial_p_glc	1460	mean	1865	nan	6.46	0.9	5.6	6.2	7.1
arterial_p_glc	30	minimum	104	nan	6.02	0.99	5.2	5.8	6.6
arterial_p_glc	365	latest	124	nan	6.41	0.95	5.4	6.1	7.1
benzodiazepine_related_sleeping_agents	730	maximum	2	0	0.17	0.83	0	0	0
benzodiazepine_related_sleeping_agents	30	maximum	2	0	0.03	0.97	0	0	0
benzodiazepine_related_sleeping_agents	180	maximum	2	0	0.09	0.91	0	0	0
benzodiazepine_related_sleeping_agents	365	maximum	2	0	0.13	0.87	0	0	0
benzodiazepine_related_sleeping_agents	1095	maximum	2	0	0.2	0.8	0	0	0
benzodiazepine_related_sleeping_agents	1095	minimum	2	0	0.2	0.8	0	0	0
benzodiazepine_related_sleeping_agents	1460	maximum	2	0	0.21	0.79	0	0	0
benzodiazepine_related_sleeping_agents	180	minimum	2	0	0.09	0.91	0	0	0
benzodiazepine_related_sleeping_agents	365	minimum	2	0	0.13	0.87	0	0	0
benzodiazepine_related_sleeping_agents	730	minimum	2	0	0.17	0.83	0	0	0
benzodiazepine_related_sleeping_agents	1825	maximum	2	0	0.22	0.78	0	0	0
benzodiazepine_related_sleeping_agents	1460	minimum	2	0	0.21	0.79	0	0	0
benzodiazepine_related_sleeping_agents	180	mean	2	0	0.09	0.91	0	0	0

benzodiazepine_related_sleeping_agents	30	minimum	2	0	0.03	0.97	0	0	0
benzodiazepine_related_sleeping_agents	30	mean	2	0	0.03	0.97	0	0	0
benzodiazepine_related_sleeping_agents	1825	latest	2	0	0.22	0.78	0	0	0
benzodiazepine_related_sleeping_agents	1460	latest	2	0	0.21	0.79	0	0	0
benzodiazepine_related_sleeping_agents	1095	latest	2	0	0.2	0.8	0	0	0
benzodiazepine_related_sleeping_agents	1825	minimum	2	0	0.22	0.78	0	0	0
benzodiazepine_related_sleeping_agents	730	latest	2	0	0.17	0.83	0	0	0
benzodiazepine_related_sleeping_agents	365	latest	2	0	0.13	0.87	0	0	0
benzodiazepine_related_sleeping_agents	1460	mean	2	0	0.21	0.79	0	0	0
benzodiazepine_related_sleeping_agents	30	latest	2	0	0.03	0.97	0	0	0
benzodiazepine_related_sleeping_agents	1825	mean	2	0	0.22	0.78	0	0	0
benzodiazepine_related_sleeping_agents	1095	mean	2	0	0.2	0.8	0	0	0
benzodiazepine_related_sleeping_agents	730	mean	2	0	0.17	0.83	0	0	0
benzodiazepine_related_sleeping_agents	365	mean	2	0	0.13	0.87	0	0	0
benzodiazepine_related_sleeping_agents	180	latest	2	0	0.09	0.91	0	0	0
benzodiazepines	30	maximum	2	0	0.03	0.97	0	0	0
benzodiazepines	180	maximum	2	0	0.1	0.9	0	0	0

benzodiazepines	730	maximum	2	0	0.19	0.81	0	0	0
benzodiazepines	1095	maximum	2	0	0.21	0.79	0	0	0
benzodiazepines	1460	maximum	2	0	0.23	0.77	0	0	0
benzodiazepines	1460	latest	2	0	0.23	0.77	0	0	0
benzodiazepines	730	minimum	2	0	0.19	0.81	0	0	0
benzodiazepines	1825	maximum	2	0	0.24	0.76	0	0	0
benzodiazepines	1095	minimum	2	0	0.21	0.79	0	0	0
benzodiazepines	180	minimum	2	0	0.1	0.9	0	0	0
benzodiazepines	365	minimum	2	0	0.15	0.85	0	0	0
benzodiazepines	1825	minimum	2	0	0.24	0.76	0	0	0
benzodiazepines	30	minimum	2	0	0.03	0.97	0	0	0
benzodiazepines	1460	minimum	2	0	0.23	0.77	0	0	0
benzodiazepines	730	latest	2	0	0.19	0.81	0	0	0
benzodiazepines	30	mean	2	0	0.03	0.97	0	0	0
benzodiazepines	30	latest	2	0	0.03	0.97	0	0	0
benzodiazepines	180	mean	2	0	0.1	0.9	0	0	0
benzodiazepines	1095	latest	2	0	0.21	0.79	0	0	0
benzodiazepines	365	latest	2	0	0.15	0.85	0	0	0
benzodiazepines	180	latest	2	0	0.1	0.9	0	0	0
benzodiazepines	365	maximum	2	0	0.15	0.85	0	0	0
benzodiazepines	1825	mean	2	0	0.24	0.76	0	0	0
benzodiazepines	1095	mean	2	0	0.21	0.79	0	0	0
benzodiazepines	1825	latest	2	0	0.24	0.76	0	0	0
benzodiazepines	1460	mean	2	0	0.23	0.77	0	0	0
benzodiazepines	365	mean	2	0	0.15	0.85	0	0	0
benzodiazepines	730	mean	2	0	0.19	0.81	0	0	0
bmi	1825	latest	520	nan	26.36	0.22	21.6	25.2	29.8
bmi	1095	latest	520	nan	26.4	0.27	21.6	25.2	29.8

bmi	1460	latest	520	nan	26.38	0.24	21.6	25.2	29.8
bmi	730	latest	519	nan	26.41	0.32	21.6	25.2	29.9
bmi	365	latest	517	nan	26.38	0.44	21.6	25.2	29.9
bmi	180	latest	515	nan	26.22	0.59	21.3	25	29.8
bmi	30	latest	499	nan	24.89	0.87	20	23.5	28.5
clozapine	30	latest	2	0	0	1	0	0	0
clozapine	1460	minimum	2	0	0.02	0.98	0	0	0
clozapine	1825	latest	2	0	0.02	0.98	0	0	0
clozapine	1460	latest	2	0	0.02	0.98	0	0	0
clozapine	1095	latest	2	0	0.02	0.98	0	0	0
clozapine	730	latest	2	0	0.02	0.98	0	0	0
clozapine	365	latest	2	0	0.01	0.99	0	0	0
clozapine	180	latest	2	0	0.01	0.99	0	0	0
clozapine	1460	mean	2	0	0.02	0.98	0	0	0
clozapine	1095	mean	2	0	0.02	0.98	0	0	0
clozapine	365	mean	2	0	0.01	0.99	0	0	0
clozapine	180	mean	2	0	0.01	0.99	0	0	0
clozapine	30	mean	2	0	0	1	0	0	0
clozapine	1825	mean	2	0	0.02	0.98	0	0	0
clozapine	730	mean	2	0	0.02	0.98	0	0	0
clozapine	1825	minimum	2	0	0.02	0.98	0	0	0
clozapine	365	minimum	2	0	0.01	0.99	0	0	0
clozapine	180	minimum	2	0	0.01	0.99	0	0	0
clozapine	30	minimum	2	0	0	1	0	0	0
clozapine	180	maximum	2	0	0.01	0.99	0	0	0
clozapine	1095	maximum	2	0	0.02	0.98	0	0	0
clozapine	1825	maximum	2	0	0.02	0.98	0	0	0
clozapine	365	maximum	2	0	0.01	0.99	0	0	0

clozapine	730	minimum	2	0	0.02	0.98	0	0	0
clozapine	30	maximum	2	0	0	1	0	0	0
clozapine	1460	maximum	2	0	0.02	0.98	0	0	0
clozapine	1095	minimum	2	0	0.02	0.98	0	0	0
clozapine	730	maximum	2	0	0.02	0.98	0	0	0
crp	730	minimum	867	nan	2.96	0.19	0.7	1.6	2.7
crp	365	minimum	913	nan	3.45	0.31	0.7	2.3	2.7
crp	1095	latest	1462	nan	5.15	0.15	0.7	2.7	4.1
crp	180	minimum	960	nan	3.88	0.49	0.7	2.7	3.1
crp	30	minimum	957	nan	4.82	0.86	0.9	2.7	4.1
crp	1825	maximum	3053	nan	22.81	0.12	1.8	3.7	14.1
crp	730	maximum	2807	nan	15.82	0.19	1.4	2.7	8.9
crp	1095	maximum	2940	nan	18.95	0.15	1.6	3	10.9
crp	365	maximum	2521	nan	11.68	0.31	1.2	2.7	6.6
crp	180	maximum	2292	nan	9.47	0.49	1.1	2.7	5.6
crp	1460	latest	1480	nan	5.18	0.13	0.7	2.7	4.1
crp	1095	minimum	838	nan	2.67	0.15	0.6	1.2	2.7
crp	1460	maximum	3012	nan	21.22	0.13	1.7	3.4	12.6
crp	1460	minimum	816	nan	2.48	0.13	0.5	1	2.7
crp	1095	mean	45240	nan	7.85	0.15	1.1	2.7	6.1
crp	180	mean	16737	nan	6.1	0.49	1	2.7	4.7
crp	30	maximum	1671	nan	7.68	0.86	1.1	2.7	4.9
crp	30	mean	5346	nan	6.12	0.86	1	2.7	4.6
crp	1825	latest	1484	nan	5.18	0.12	0.7	2.7	4.1
crp	730	latest	1434	nan	5.09	0.19	0.8	2.7	4.1
crp	365	latest	1360	nan	5.02	0.31	0.8	2.7	4.1
crp	1825	minimum	793	nan	2.34	0.12	0.4	0.9	2.7
crp	30	latest	1143	nan	5.56	0.86	1	2.7	4.5

crp	1825	mean	52604	nan	8.39	0.12	1.2	2.7	6.7
crp	1460	mean	50002	nan	8.21	0.13	1.2	2.7	6.5
crp	730	mean	38051	nan	7.3	0.19	1.1	2.7	5.6
crp	365	mean	25833	nan	6.49	0.31	1.1	2.7	5
crp	180	latest	1300	nan	5.04	0.49	0.9	2.7	4.1
diuretics	365	mean	2	0	0.02	0.98	0	0	0
diuretics	730	minimum	2	0	0.03	0.97	0	0	0
diuretics	365	maximum	2	0	0.02	0.98	0	0	0
diuretics	1095	minimum	2	0	0.03	0.97	0	0	0
diuretics	30	maximum	2	0	0	1	0	0	0
diuretics	730	maximum	2	0	0.03	0.97	0	0	0
diuretics	1460	maximum	2	0	0.03	0.97	0	0	0
diuretics	30	minimum	2	0	0	1	0	0	0
diuretics	180	minimum	2	0	0.01	0.99	0	0	0
diuretics	365	minimum	2	0	0.02	0.98	0	0	0
diuretics	1460	minimum	2	0	0.03	0.97	0	0	0
diuretics	1095	maximum	2	0	0.03	0.97	0	0	0
diuretics	1825	minimum	2	0	0.04	0.96	0	0	0
diuretics	1825	maximum	2	0	0.04	0.96	0	0	0
diuretics	180	mean	2	0	0.01	0.99	0	0	0
diuretics	1460	latest	2	0	0.03	0.97	0	0	0
diuretics	1825	latest	2	0	0.04	0.96	0	0	0
diuretics	1095	latest	2	0	0.03	0.97	0	0	0
diuretics	730	latest	2	0	0.03	0.97	0	0	0
diuretics	180	latest	2	0	0.01	0.99	0	0	0
diuretics	180	maximum	2	0	0.01	0.99	0	0	0
diuretics	30	mean	2	0	0	1	0	0	0
diuretics	1825	mean	2	0	0.04	0.96	0	0	0

diuretics	1460	mean	2	0	0.03	0.97	0	0	0
diuretics	1095	mean	2	0	0.03	0.97	0	0	0
diuretics	730	mean	2	0	0.03	0.97	0	0	0
diuretics	365	latest	2	0	0.02	0.98	0	0	0
diuretics	30	latest	2	0	0	1	0	0	0
egfr	730	minimum	162	nan	105.44	0.09	80	108	135
egfr	365	minimum	167	nan	108.96	0.18	83	108	135
egfr	180	minimum	163	nan	110.8	0.35	84	135	135
egfr	30	minimum	150	nan	111.89	0.8	85	135	135
egfr	1825	maximum	220	nan	123.27	0.06	108	135	135
egfr	30	maximum	153	nan	114.03	0.8	87	135	135
egfr	1460	latest	174	nan	114.81	0.06	88	135	135
egfr	730	maximum	213	nan	121.3	0.09	108	135	135
egfr	30	mean	1645	nan	113.01	0.8	86.5	135	135
egfr	1825	latest	174	nan	114.8	0.06	88	135	135
egfr	1460	maximum	220	nan	122.93	0.06	108	135	135
egfr	1095	minimum	161	nan	102.68	0.07	78	108	135
egfr	180	maximum	187	nan	117.18	0.35	102	135	135
egfr	1095	maximum	211	nan	122.35	0.07	108	135	135
egfr	1460	minimum	161	nan	100.64	0.06	77	99	135
egfr	365	maximum	197	nan	119.16	0.18	108	135	135
egfr	1825	minimum	160	nan	99.02	0.06	77	90	135
egfr	730	latest	174	nan	114.84	0.09	88	135	135
egfr	1095	latest	174	nan	114.83	0.07	88	135	135
egfr	365	latest	171	nan	114.75	0.18	88	135	135
egfr	30	latest	151	nan	113.15	0.8	86	135	135
egfr	180	latest	167	nan	114.36	0.35	87	135	135
egfr	1460	mean	22825	nan	112.97	0.06	97.4	118	135

egfr	1095	mean	19821	nan	113.49	0.07	97.8	119.5	135
egfr	730	mean	15869	nan	114.06	0.09	97.8	121.5	135
egfr	365	mean	10031	nan	114.44	0.18	96	128.2	135
egfr	180	mean	6058	nan	114.2	0.35	90	135	135
egfr	1825	mean	24949	nan	112.52	0.06	97	117	135
essential_hypertension	1095	maximum	2	0	0.02	0.98	0	0	0
essential_hypertension	30	minimum	2	0	0	1	0	0	0
essential_hypertension	1825	maximum	2	0	0.02	0.98	0	0	0
essential_hypertension	1460	maximum	2	0	0.02	0.98	0	0	0
essential_hypertension	730	maximum	2	0	0.01	0.99	0	0	0
essential_hypertension	180	minimum	2	0	0.01	0.99	0	0	0
essential_hypertension	180	maximum	2	0	0.01	0.99	0	0	0
essential_hypertension	30	maximum	2	0	0	1	0	0	0
essential_hypertension	1095	latest	2	0	0.02	0.98	0	0	0
essential_hypertension	1460	minimum	2	0	0.02	0.98	0	0	0
essential_hypertension	1825	latest	2	0	0.02	0.98	0	0	0
essential_hypertension	365	maximum	2	0	0.01	0.99	0	0	0
essential_hypertension	365	minimum	2	0	0.01	0.99	0	0	0
essential_hypertension	730	latest	2	0	0.01	0.99	0	0	0
essential_hypertension	1095	minimum	2	0	0.02	0.98	0	0	0
essential_hypertension	365	latest	2	0	0.01	0.99	0	0	0
essential_hypertension	180	latest	2	0	0.01	0.99	0	0	0
essential_hypertension	730	minimum	2	0	0.01	0.99	0	0	0
essential_hypertension	30	latest	2	0	0	1	0	0	0
essential_hypertension	1825	mean	2	0	0.02	0.98	0	0	0
essential_hypertension	1460	mean	2	0	0.02	0.98	0	0	0
essential_hypertension	1460	latest	2	0	0.02	0.98	0	0	0
essential_hypertension	730	mean	2	0	0.01	0.99	0	0	0

essential_hypertension	365	mean	2	0	0.01	0.99	0	0	0
essential_hypertension	180	mean	2	0	0.01	0.99	0	0	0
essential_hypertension	30	mean	2	0	0	1	0	0	0
essential_hypertension	1825	minimum	2	0	0.02	0.98	0	0	0
essential_hypertension	1095	mean	2	0	0.02	0.98	0	0	0
f0 - Organic disorders	30	maximum	2	0	0	1	0	0	0
f0 - Organic disorders	180	maximum	2	0	0.01	0.99	0	0	0
f0 - Organic disorders	365	maximum	2	0	0.02	0.98	0	0	0
f0 - Organic disorders	730	maximum	2	0	0.02	0.98	0	0	0
f0 - Organic disorders	1095	maximum	2	0	0.03	0.97	0	0	0
f0 - Organic disorders	30	minimum	2	0	0	1	0	0	0
f0 - Organic disorders	1825	maximum	2	0	0.03	0.97	0	0	0
f0 - Organic disorders	180	minimum	2	0	0.01	0.99	0	0	0
f0 - Organic disorders	730	minimum	2	0	0.02	0.98	0	0	0
f0 - Organic disorders	1095	minimum	2	0	0.03	0.97	0	0	0
f0 - Organic disorders	30	mean	2	0	0	1	0	0	0
f0 - Organic disorders	1460	maximum	2	0	0.03	0.97	0	0	0
f0 - Organic disorders	180	mean	2	0	0.01	0.99	0	0	0
f0 - Organic disorders	1825	minimum	2	0	0.03	0.97	0	0	0
f0 - Organic disorders	730	mean	2	0	0.02	0.98	0	0	0
f0 - Organic disorders	365	minimum	2	0	0.02	0.98	0	0	0
f0 - Organic disorders	1460	minimum	2	0	0.03	0.97	0	0	0
f0 - Organic disorders	1825	latest	2	0	0.03	0.97	0	0	0
f0 - Organic disorders	1460	latest	2	0	0.03	0.97	0	0	0
f0 - Organic disorders	1095	latest	2	0	0.03	0.97	0	0	0
f0 - Organic disorders	730	latest	2	0	0.02	0.98	0	0	0
f0 - Organic disorders	365	mean	2	0	0.02	0.98	0	0	0
f0 - Organic disorders	180	latest	2	0	0.01	0.99	0	0	0

f0 - Organic disorders	30	latest	2	0	0	1	0	0	0
f0 - Organic disorders	1825	mean	2	0	0.03	0.97	0	0	0
f0 - Organic disorders	1460	mean	2	0	0.03	0.97	0	0	0
f0 - Organic disorders	1095	mean	2	0	0.03	0.97	0	0	0
f0 - Organic disorders	365	latest	2	0	0.02	0.98	0	0	0
f1 - Substance abuse	1095	minimum	2	0	0.12	0.88	0	0	0
f1 - Substance abuse	730	minimum	2	0	0.11	0.89	0	0	0
f1 - Substance abuse	365	minimum	2	0	0.09	0.91	0	0	0
f1 - Substance abuse	180	minimum	2	0	0.06	0.94	0	0	0
f1 - Substance abuse	30	minimum	2	0	0.03	0.97	0	0	0
f1 - Substance abuse	1825	maximum	2	0	0.14	0.86	0	0	0
f1 - Substance abuse	730	maximum	2	0	0.11	0.89	0	0	0
f1 - Substance abuse	1095	maximum	2	0	0.12	0.88	0	0	0
f1 - Substance abuse	365	maximum	2	0	0.09	0.91	0	0	0
f1 - Substance abuse	180	maximum	2	0	0.06	0.94	0	0	0
f1 - Substance abuse	30	maximum	2	0	0.03	0.97	0	0	0
f1 - Substance abuse	180	mean	2	0	0.06	0.94	0	0	0
f1 - Substance abuse	1460	maximum	2	0	0.13	0.87	0	0	0
f1 - Substance abuse	1460	minimum	2	0	0.13	0.87	0	0	0
f1 - Substance abuse	1825	minimum	2	0	0.14	0.86	0	0	0
f1 - Substance abuse	1460	latest	2	0	0.13	0.87	0	0	0
f1 - Substance abuse	365	mean	2	0	0.09	0.91	0	0	0
f1 - Substance abuse	1825	latest	2	0	0.14	0.86	0	0	0
f1 - Substance abuse	730	mean	2	0	0.11	0.89	0	0	0
f1 - Substance abuse	1460	mean	2	0	0.13	0.87	0	0	0
f1 - Substance abuse	1825	mean	2	0	0.14	0.86	0	0	0
f1 - Substance abuse	1095	mean	2	0	0.12	0.88	0	0	0
f1 - Substance abuse	180	latest	2	0	0.06	0.94	0	0	0

f1 - Substance abuse	365	latest	2	0	0.09	0.91	0	0	0
f1 - Substance abuse	730	latest	2	0	0.11	0.89	0	0	0
f1 - Substance abuse	1095	latest	2	0	0.12	0.88	0	0	0
f1 - Substance abuse	30	mean	2	0	0.03	0.97	0	0	0
f1 - Substance abuse	30	latest	2	0	0.03	0.97	0	0	0
f2 - Psychotic disorders	730	latest	2	0	0.16	0.84	0	0	0
f2 - Psychotic disorders	1825	mean	2	0	0.18	0.82	0	0	0
f2 - Psychotic disorders	30	latest	2	0	0.06	0.94	0	0	0
f2 - Psychotic disorders	180	latest	2	0	0.11	0.89	0	0	0
f2 - Psychotic disorders	365	latest	2	0	0.13	0.87	0	0	0
f2 - Psychotic disorders	1460	minimum	2	0	0.17	0.83	0	0	0
f2 - Psychotic disorders	180	maximum	2	0	0.11	0.89	0	0	0
f2 - Psychotic disorders	1825	latest	2	0	0.18	0.82	0	0	0
f2 - Psychotic disorders	365	maximum	2	0	0.13	0.87	0	0	0
f2 - Psychotic disorders	30	maximum	2	0	0.06	0.94	0	0	0
f2 - Psychotic disorders	1460	latest	2	0	0.17	0.83	0	0	0
f2 - Psychotic disorders	1460	mean	2	0	0.17	0.83	0	0	0
f2 - Psychotic disorders	1095	latest	2	0	0.17	0.83	0	0	0
f2 - Psychotic disorders	1095	mean	2	0	0.17	0.83	0	0	0
f2 - Psychotic disorders	730	mean	2	0	0.16	0.84	0	0	0
f2 - Psychotic disorders	365	mean	2	0	0.13	0.87	0	0	0
f2 - Psychotic disorders	180	minimum	2	0	0.11	0.89	0	0	0
f2 - Psychotic disorders	30	mean	2	0	0.06	0.94	0	0	0
f2 - Psychotic disorders	1825	maximum	2	0	0.18	0.82	0	0	0
f2 - Psychotic disorders	730	minimum	2	0	0.16	0.84	0	0	0
f2 - Psychotic disorders	1460	maximum	2	0	0.17	0.83	0	0	0
f2 - Psychotic disorders	365	minimum	2	0	0.13	0.87	0	0	0
f2 - Psychotic disorders	730	maximum	2	0	0.16	0.84	0	0	0

f2 - Psychotic disorders	30	minimum	2	0	0.06	0.94	0	0	0
f2 - Psychotic disorders	1095	minimum	2	0	0.17	0.83	0	0	0
f2 - Psychotic disorders	180	mean	2	0	0.11	0.89	0	0	0
f2 - Psychotic disorders	1825	minimum	2	0	0.18	0.82	0	0	0
f2 - Psychotic disorders	1095	maximum	2	0	0.17	0.83	0	0	0
f3 - Mood disorders	730	mean	2	0	0.39	0.61	0	0	1
f3 - Mood disorders	1095	mean	2	0	0.42	0.58	0	0	1
f3 - Mood disorders	730	maximum	2	0	0.39	0.61	0	0	1
f3 - Mood disorders	1825	mean	2	0	0.45	0.55	0	0	1
f3 - Mood disorders	1460	mean	2	0	0.44	0.56	0	0	1
f3 - Mood disorders	1095	latest	2	0	0.42	0.58	0	0	1
f3 - Mood disorders	1460	latest	2	0	0.44	0.56	0	0	1
f3 - Mood disorders	365	latest	2	0	0.32	0.68	0	0	1
f3 - Mood disorders	180	latest	2	0	0.25	0.75	0	0	0
f3 - Mood disorders	730	latest	2	0	0.39	0.61	0	0	1
f3 - Mood disorders	30	maximum	2	0	0.12	0.88	0	0	0
f3 - Mood disorders	1825	latest	2	0	0.45	0.55	0	0	1
f3 - Mood disorders	180	maximum	2	0	0.25	0.75	0	0	0
f3 - Mood disorders	30	latest	2	0	0.12	0.88	0	0	0
f3 - Mood disorders	1095	maximum	2	0	0.42	0.58	0	0	1
f3 - Mood disorders	365	mean	2	0	0.32	0.68	0	0	1
f3 - Mood disorders	180	mean	2	0	0.25	0.75	0	0	0
f3 - Mood disorders	30	mean	2	0	0.12	0.88	0	0	0
f3 - Mood disorders	1825	minimum	2	0	0.45	0.55	0	0	1
f3 - Mood disorders	1460	minimum	2	0	0.44	0.56	0	0	1
f3 - Mood disorders	1095	minimum	2	0	0.42	0.58	0	0	1
f3 - Mood disorders	365	maximum	2	0	0.32	0.68	0	0	1
f3 - Mood disorders	365	minimum	2	0	0.32	0.68	0	0	1

f3 - Mood disorders	180	minimum	2	0	0.25	0.75	0	0	0
f3 - Mood disorders	30	minimum	2	0	0.12	0.88	0	0	0
f3 - Mood disorders	1825	maximum	2	0	0.45	0.55	0	0	1
f3 - Mood disorders	1460	maximum	2	0	0.44	0.56	0	0	1
f3 - Mood disorders	730	minimum	2	0	0.39	0.61	0	0	1
f4 - Neurotic & stress-related	180	maximum	2	0	0.19	0.81	0	0	0
f4 - Neurotic & stress-related	365	maximum	2	0	0.26	0.74	0	0	1
f4 - Neurotic & stress-related	1825	minimum	2	0	0.4	0.6	0	0	1
f4 - Neurotic & stress-related	730	maximum	2	0	0.33	0.67	0	0	1
f4 - Neurotic & stress-related	1095	maximum	2	0	0.36	0.64	0	0	1
f4 - Neurotic & stress-related	1095	latest	2	0	0.36	0.64	0	0	1
f4 - Neurotic & stress-related	30	minimum	2	0	0.09	0.91	0	0	0
f4 - Neurotic & stress-related	1095	minimum	2	0	0.36	0.64	0	0	1
f4 - Neurotic & stress-related	180	minimum	2	0	0.19	0.81	0	0	0
f4 - Neurotic & stress-related	730	minimum	2	0	0.33	0.67	0	0	1
f4 - Neurotic & stress-related	365	minimum	2	0	0.26	0.74	0	0	1
f4 - Neurotic & stress-related	30	maximum	2	0	0.09	0.91	0	0	0
f4 - Neurotic & stress-related	1825	maximum	2	0	0.4	0.6	0	0	1
f4 - Neurotic & stress-related	1460	maximum	2	0	0.38	0.62	0	0	1
f4 - Neurotic & stress-related	1460	minimum	2	0	0.38	0.62	0	0	1
f4 - Neurotic & stress-related	30	mean	2	0	0.09	0.91	0	0	0
f4 - Neurotic & stress-related	1460	mean	2	0	0.38	0.62	0	0	1
f4 - Neurotic & stress-related	365	latest	2	0	0.26	0.74	0	0	1
f4 - Neurotic & stress-related	1460	latest	2	0	0.38	0.62	0	0	1
f4 - Neurotic & stress-related	1825	latest	2	0	0.4	0.6	0	0	1
f4 - Neurotic & stress-related	30	latest	2	0	0.09	0.91	0	0	0
f4 - Neurotic & stress-related	180	latest	2	0	0.19	0.81	0	0	0
f4 - Neurotic & stress-related	730	latest	2	0	0.33	0.67	0	0	1

f4 - Neurotic & stress-related	1095	mean	2	0	0.36	0.64	0	0	1
f4 - Neurotic & stress-related	730	mean	2	0	0.33	0.67	0	0	1
f4 - Neurotic & stress-related	365	mean	2	0	0.26	0.74	0	0	1
f4 - Neurotic & stress-related	180	mean	2	0	0.19	0.81	0	0	0
f4 - Neurotic & stress-related	1825	mean	2	0	0.4	0.6	0	0	1
f5 - Eating & sleeping disorders	1460	mean	2	0	0.07	0.93	0	0	0
f5 - Eating & sleeping disorders	180	latest	2	0	0.04	0.96	0	0	0
f5 - Eating & sleeping disorders	30	latest	2	0	0.02	0.98	0	0	0
f5 - Eating & sleeping disorders	1825	mean	2	0	0.07	0.93	0	0	0
f5 - Eating & sleeping disorders	1095	minimum	2	0	0.06	0.94	0	0	0
f5 - Eating & sleeping disorders	365	latest	2	0	0.05	0.95	0	0	0
f5 - Eating & sleeping disorders	180	mean	2	0	0.04	0.96	0	0	0
f5 - Eating & sleeping disorders	1825	minimum	2	0	0.07	0.93	0	0	0
f5 - Eating & sleeping disorders	1825	latest	2	0	0.07	0.93	0	0	0
f5 - Eating & sleeping disorders	30	mean	2	0	0.02	0.98	0	0	0
f5 - Eating & sleeping disorders	1460	minimum	2	0	0.07	0.93	0	0	0
f5 - Eating & sleeping disorders	730	mean	2	0	0.06	0.94	0	0	0
f5 - Eating & sleeping disorders	730	latest	2	0	0.06	0.94	0	0	0
f5 - Eating & sleeping disorders	1095	mean	2	0	0.06	0.94	0	0	0
f5 - Eating & sleeping disorders	1460	latest	2	0	0.07	0.93	0	0	0
f5 - Eating & sleeping disorders	730	minimum	2	0	0.06	0.94	0	0	0
f5 - Eating & sleeping disorders	30	maximum	2	0	0.02	0.98	0	0	0
f5 - Eating & sleeping disorders	180	maximum	2	0	0.04	0.96	0	0	0
f5 - Eating & sleeping disorders	1095	maximum	2	0	0.06	0.94	0	0	0
f5 - Eating & sleeping disorders	730	maximum	2	0	0.06	0.94	0	0	0
f5 - Eating & sleeping disorders	1460	maximum	2	0	0.07	0.93	0	0	0
f5 - Eating & sleeping disorders	1095	latest	2	0	0.06	0.94	0	0	0
f5 - Eating & sleeping disorders	30	minimum	2	0	0.02	0.98	0	0	0

f5 - Eating & sleeping disorders	365	minimum	2	0	0.05	0.95	0	0	0
f5 - Eating & sleeping disorders	365	maximum	2	0	0.05	0.95	0	0	0
f5 - Eating & sleeping disorders	180	minimum	2	0	0.04	0.96	0	0	0
f5 - Eating & sleeping disorders	365	mean	2	0	0.05	0.95	0	0	0
f5 - Eating & sleeping disorders	1825	maximum	2	0	0.07	0.93	0	0	0
f6 - Perosnality disorders	730	latest	2	0	0.18	0.82	0	0	0
f6 - Perosnality disorders	730	maximum	2	0	0.18	0.82	0	0	0
f6 - Perosnality disorders	180	latest	2	0	0.12	0.88	0	0	0
f6 - Perosnality disorders	365	latest	2	0	0.15	0.85	0	0	0
f6 - Perosnality disorders	30	latest	2	0	0.07	0.93	0	0	0
f6 - Perosnality disorders	730	mean	2	0	0.18	0.82	0	0	0
f6 - Perosnality disorders	1095	mean	2	0	0.2	0.8	0	0	0
f6 - Perosnality disorders	365	mean	2	0	0.15	0.85	0	0	0
f6 - Perosnality disorders	180	mean	2	0	0.12	0.88	0	0	0
f6 - Perosnality disorders	30	mean	2	0	0.07	0.93	0	0	0
f6 - Perosnality disorders	1095	latest	2	0	0.2	0.8	0	0	0
f6 - Perosnality disorders	1825	mean	2	0	0.21	0.79	0	0	0
f6 - Perosnality disorders	1825	latest	2	0	0.21	0.79	0	0	0
f6 - Perosnality disorders	1825	minimum	2	0	0.21	0.79	0	0	0
f6 - Perosnality disorders	30	maximum	2	0	0.07	0.93	0	0	0
f6 - Perosnality disorders	1460	mean	2	0	0.2	0.8	0	0	0
f6 - Perosnality disorders	1460	minimum	2	0	0.2	0.8	0	0	0
f6 - Perosnality disorders	1095	minimum	2	0	0.2	0.8	0	0	0
f6 - Perosnality disorders	730	minimum	2	0	0.18	0.82	0	0	0
f6 - Perosnality disorders	365	minimum	2	0	0.15	0.85	0	0	0
f6 - Perosnality disorders	180	minimum	2	0	0.12	0.88	0	0	0
f6 - Perosnality disorders	1460	latest	2	0	0.2	0.8	0	0	0
f6 - Perosnality disorders	1825	maximum	2	0	0.21	0.79	0	0	0

f6 - Perosnality disorders	1460	maximum	2	0	0.2	0.8	0	0	0
f6 - Perosnality disorders	1095	maximum	2	0	0.2	0.8	0	0	0
f6 - Perosnality disorders	365	maximum	2	0	0.15	0.85	0	0	0
f6 - Perosnality disorders	180	maximum	2	0	0.12	0.88	0	0	0
f6 - Perosnality disorders	30	minimum	2	0	0.07	0.93	0	0	0
f7 - Mental retardation	1095	maximum	2	0	0.01	0.99	0	0	0
f7 - Mental retardation	365	maximum	2	0	0.01	0.99	0	0	0
f7 - Mental retardation	180	maximum	2	0	0.01	0.99	0	0	0
f7 - Mental retardation	30	maximum	2	0	0	1	0	0	0
f7 - Mental retardation	1825	minimum	2	0	0.02	0.98	0	0	0
f7 - Mental retardation	30	mean	2	0	0	1	0	0	0
f7 - Mental retardation	730	mean	2	0	0.01	0.99	0	0	0
f7 - Mental retardation	365	mean	2	0	0.01	0.99	0	0	0
f7 - Mental retardation	1460	maximum	2	0	0.02	0.98	0	0	0
f7 - Mental retardation	1095	mean	2	0	0.01	0.99	0	0	0
f7 - Mental retardation	1460	mean	2	0	0.02	0.98	0	0	0
f7 - Mental retardation	730	maximum	2	0	0.01	0.99	0	0	0
f7 - Mental retardation	180	mean	2	0	0.01	0.99	0	0	0
f7 - Mental retardation	1825	mean	2	0	0.02	0.98	0	0	0
f7 - Mental retardation	1825	maximum	2	0	0.02	0.98	0	0	0
f7 - Mental retardation	1825	latest	2	0	0.02	0.98	0	0	0
f7 - Mental retardation	30	latest	2	0	0	1	0	0	0
f7 - Mental retardation	180	minimum	2	0	0.01	0.99	0	0	0
f7 - Mental retardation	365	minimum	2	0	0.01	0.99	0	0	0
f7 - Mental retardation	730	minimum	2	0	0.01	0.99	0	0	0
f7 - Mental retardation	1460	minimum	2	0	0.02	0.98	0	0	0
f7 - Mental retardation	1095	minimum	2	0	0.01	0.99	0	0	0
f7 - Mental retardation	180	latest	2	0	0.01	0.99	0	0	0

f7 - Mental retardation	365	latest	2	0	0.01	0.99	0	0	0
f7 - Mental retardation	730	latest	2	0	0.01	0.99	0	0	0
f7 - Mental retardation	30	minimum	2	0	0	1	0	0	0
f7 - Mental retardation	1460	latest	2	0	0.02	0.98	0	0	0
f7 - Mental retardation	1095	latest	2	0	0.01	0.99	0	0	0
f8 - Developmental disorders	1095	latest	2	0	0.05	0.95	0	0	0
f8 - Developmental disorders	180	latest	2	0	0.02	0.98	0	0	0
f8 - Developmental disorders	1460	minimum	2	0	0.05	0.95	0	0	0
f8 - Developmental disorders	730	latest	2	0	0.04	0.96	0	0	0
f8 - Developmental disorders	180	minimum	2	0	0.02	0.98	0	0	0
f8 - Developmental disorders	30	latest	2	0	0.01	0.99	0	0	0
f8 - Developmental disorders	365	mean	2	0	0.03	0.97	0	0	0
f8 - Developmental disorders	1095	minimum	2	0	0.05	0.95	0	0	0
f8 - Developmental disorders	730	minimum	2	0	0.04	0.96	0	0	0
f8 - Developmental disorders	365	minimum	2	0	0.03	0.97	0	0	0
f8 - Developmental disorders	365	latest	2	0	0.03	0.97	0	0	0
f8 - Developmental disorders	1825	latest	2	0	0.05	0.95	0	0	0
f8 - Developmental disorders	1825	mean	2	0	0.05	0.95	0	0	0
f8 - Developmental disorders	1460	latest	2	0	0.05	0.95	0	0	0
f8 - Developmental disorders	1095	mean	2	0	0.05	0.95	0	0	0
f8 - Developmental disorders	730	maximum	2	0	0.04	0.96	0	0	0
f8 - Developmental disorders	365	maximum	2	0	0.03	0.97	0	0	0
f8 - Developmental disorders	1095	maximum	2	0	0.05	0.95	0	0	0
f8 - Developmental disorders	30	maximum	2	0	0.01	0.99	0	0	0
f8 - Developmental disorders	180	maximum	2	0	0.02	0.98	0	0	0
f8 - Developmental disorders	1460	maximum	2	0	0.05	0.95	0	0	0
f8 - Developmental disorders	1460	mean	2	0	0.05	0.95	0	0	0
f8 - Developmental disorders	30	minimum	2	0	0.01	0.99	0	0	0

f8 - Developmental disorders	1825	maximum	2	0	0.05	0.95	0	0	0
f8 - Developmental disorders	30	mean	2	0	0.01	0.99	0	0	0
f8 - Developmental disorders	1825	minimum	2	0	0.05	0.95	0	0	0
f8 - Developmental disorders	730	mean	2	0	0.04	0.96	0	0	0
f8 - Developmental disorders	180	mean	2	0	0.02	0.98	0	0	0
f9 - Child & adolescent disorders	1095	latest	2	0	0.11	0.89	0	0	0
f9 - Child & adolescent disorders	730	latest	2	0	0.1	0.9	0	0	0
f9 - Child & adolescent disorders	365	latest	2	0	0.08	0.92	0	0	0
f9 - Child & adolescent disorders	1460	latest	2	0	0.12	0.88	0	0	0
f9 - Child & adolescent disorders	730	maximum	2	0	0.1	0.9	0	0	0
f9 - Child & adolescent disorders	365	maximum	2	0	0.08	0.92	0	0	0
f9 - Child & adolescent disorders	1825	latest	2	0	0.13	0.87	0	0	0
f9 - Child & adolescent disorders	1095	maximum	2	0	0.11	0.89	0	0	0
f9 - Child & adolescent disorders	1460	maximum	2	0	0.12	0.88	0	0	0
f9 - Child & adolescent disorders	30	maximum	2	0	0.03	0.97	0	0	0
f9 - Child & adolescent disorders	180	maximum	2	0	0.06	0.94	0	0	0
f9 - Child & adolescent disorders	30	latest	2	0	0.03	0.97	0	0	0
f9 - Child & adolescent disorders	180	minimum	2	0	0.06	0.94	0	0	0
f9 - Child & adolescent disorders	180	latest	2	0	0.06	0.94	0	0	0
f9 - Child & adolescent disorders	365	minimum	2	0	0.08	0.92	0	0	0
f9 - Child & adolescent disorders	1825	mean	2	0	0.13	0.87	0	0	0
f9 - Child & adolescent disorders	1460	mean	2	0	0.12	0.88	0	0	0
f9 - Child & adolescent disorders	30	minimum	2	0	0.03	0.97	0	0	0
f9 - Child & adolescent disorders	730	mean	2	0	0.1	0.9	0	0	0
f9 - Child & adolescent disorders	365	mean	2	0	0.08	0.92	0	0	0
f9 - Child & adolescent disorders	180	mean	2	0	0.06	0.94	0	0	0
f9 - Child & adolescent disorders	30	mean	2	0	0.03	0.97	0	0	0
f9 - Child & adolescent disorders	1825	minimum	2	0	0.13	0.87	0	0	0

f9 - Child & adolescent disorders	1095	minimum	2	0	0.11	0.89	0	0	0
f9 - Child & adolescent disorders	1825	maximum	2	0	0.13	0.87	0	0	0
f9 - Child & adolescent disorders	1095	mean	2	0	0.11	0.89	0	0	0
f9 - Child & adolescent disorders	1460	minimum	2	0	0.12	0.88	0	0	0
f9 - Child & adolescent disorders	730	minimum	2	0	0.1	0.9	0	0	0
fasting_ldl	365	latest	77	nan	2.88	0.91	2.2	2.8	3.4
fasting_ldl	1095	latest	79	nan	2.88	0.82	2.2	2.8	3.4
fasting_ldl	730	latest	79	nan	2.88	0.85	2.2	2.8	3.4
fasting_ldl	180	latest	77	nan	2.9	0.95	2.2	2.8	3.5
fasting_ldl	30	latest	74	nan	2.89	0.99	2.2	2.8	3.4
fasting_ldl	1825	mean	876	nan	2.89	0.77	2.2	2.8	3.4
fasting_ldl	1460	mean	848	nan	2.89	0.79	2.2	2.8	3.4
fasting_ldl	1095	mean	753	nan	2.89	0.82	2.2	2.8	3.4
fasting_ldl	365	mean	426	nan	2.88	0.91	2.2	2.8	3.4
fasting_ldl	180	mean	283	nan	2.9	0.95	2.2	2.8	3.5
fasting_ldl	30	mean	107	nan	2.89	0.99	2.2	2.8	3.4
fasting_ldl	730	mean	622	nan	2.89	0.85	2.2	2.8	3.4
fasting_ldl	1460	minimum	75	nan	2.76	0.79	2.1	2.7	3.3
fasting_ldl	1460	maximum	82	nan	3.02	0.79	2.3	2.9	3.6
fasting_ldl	30	maximum	74	nan	2.89	0.99	2.2	2.8	3.4
fasting_ldl	180	maximum	77	nan	2.92	0.95	2.2	2.8	3.5
fasting_ldl	365	maximum	77	nan	2.93	0.91	2.2	2.8	3.5
fasting_ldl	1095	minimum	75	nan	2.78	0.82	2.1	2.7	3.3
fasting_ldl	1095	maximum	81	nan	3	0.82	2.3	2.9	3.6
fasting_ldl	730	maximum	81	nan	2.98	0.85	2.3	2.9	3.6
fasting_ldl	1825	latest	79	nan	2.88	0.77	2.2	2.8	3.4
fasting_ldl	1825	maximum	82	nan	3.03	0.77	2.3	2.9	3.6
fasting_ldl	30	minimum	74	nan	2.89	0.99	2.2	2.8	3.4

fasting_ldl	180	minimum	76	nan	2.87	0.95	2.2	2.8	3.4
fasting_ldl	365	minimum	76	nan	2.83	0.91	2.2	2.8	3.4
fasting_ldl	730	minimum	77	nan	2.8	0.85	2.1	2.7	3.3
fasting_ldl	1460	latest	79	nan	2.88	0.79	2.2	2.8	3.4
fasting_ldl	1825	minimum	75	nan	2.75	0.77	2.1	2.7	3.3
fasting_p_glc	1460	mean	437	nan	5.4	0.82	5	5.4	5.7
fasting_p_glc	30	mean	62	nan	5.41	0.99	5	5.4	5.8
fasting_p_glc	1825	latest	40	nan	5.41	0.8	5	5.4	5.8
fasting_p_glc	1460	latest	40	nan	5.4	0.82	5	5.4	5.8
fasting_p_glc	180	latest	39	nan	5.41	0.95	5	5.4	5.8
fasting_p_glc	1095	minimum	42	nan	5.32	0.84	5	5.3	5.7
fasting_p_glc	730	minimum	41	nan	5.34	0.87	5	5.3	5.7
fasting_p_glc	365	minimum	40	nan	5.37	0.92	5	5.3	5.7
fasting_p_glc	30	minimum	39	nan	5.41	0.99	5	5.4	5.8
fasting_p_glc	1825	maximum	39	nan	5.5	0.8	5.1	5.5	5.9
fasting_p_glc	1460	maximum	39	nan	5.49	0.82	5.1	5.5	5.9
fasting_p_glc	1095	maximum	39	nan	5.48	0.84	5.1	5.4	5.8
fasting_p_glc	365	maximum	40	nan	5.44	0.92	5.1	5.4	5.8
fasting_p_glc	730	maximum	40	nan	5.46	0.87	5.1	5.4	5.8
fasting_p_glc	180	maximum	39	nan	5.43	0.95	5.1	5.4	5.8
fasting_p_glc	365	mean	236	nan	5.41	0.92	5	5.4	5.8
fasting_p_glc	730	mean	335	nan	5.4	0.87	5	5.4	5.7
fasting_p_glc	1095	mean	402	nan	5.4	0.84	5	5.4	5.7
fasting_p_glc	1460	minimum	42	nan	5.31	0.82	5	5.3	5.7
fasting_p_glc	1825	minimum	42	nan	5.3	0.8	5	5.3	5.6
fasting_p_glc	1095	latest	40	nan	5.41	0.84	5	5.4	5.8
fasting_p_glc	730	latest	40	nan	5.4	0.87	5	5.4	5.8
fasting_p_glc	365	latest	40	nan	5.4	0.92	5	5.4	5.8

fasting_p_glc	30	latest	39	nan	5.41	0.99	5	5.4	5.8
fasting_p_glc	1825	mean	451	nan	5.4	0.8	5	5.4	5.7
fasting_p_glc	180	mean	159	nan	5.41	0.95	5	5.4	5.8
fasting_p_glc	30	maximum	39	nan	5.41	0.99	5	5.4	5.8
fasting_p_glc	180	minimum	39	nan	5.39	0.95	5	5.4	5.7
gerd	1460	maximum	2	0	0.01	0.99	0	0	0
gerd	1825	latest	2	0	0.01	0.99	0	0	0
gerd	180	mean	2	0	0	1	0	0	0
gerd	1460	minimum	2	0	0.01	0.99	0	0	0
gerd	1095	minimum	2	0	0.01	0.99	0	0	0
gerd	30	maximum	2	0	0	1	0	0	0
gerd	180	maximum	2	0	0	1	0	0	0
gerd	365	maximum	2	0	0	1	0	0	0
gerd	730	maximum	2	0	0.01	0.99	0	0	0
gerd	1095	maximum	2	0	0.01	0.99	0	0	0
gerd	1825	maximum	2	0	0.01	0.99	0	0	0
gerd	30	minimum	2	0	0	1	0	0	0
gerd	180	minimum	2	0	0	1	0	0	0
gerd	365	mean	2	0	0	1	0	0	0
gerd	1825	minimum	2	0	0.01	0.99	0	0	0
gerd	1460	latest	2	0	0.01	0.99	0	0	0
gerd	1095	latest	2	0	0.01	0.99	0	0	0
gerd	730	latest	2	0	0.01	0.99	0	0	0
gerd	365	latest	2	0	0	1	0	0	0
gerd	180	latest	2	0	0	1	0	0	0
gerd	30	latest	2	0	0	1	0	0	0
gerd	1825	mean	2	0	0.01	0.99	0	0	0
gerd	1460	mean	2	0	0.01	0.99	0	0	0

gerd	1095	mean	2	0	0.01	0.99	0	0	0
gerd	730	mean	2	0	0.01	0.99	0	0	0
gerd	365	minimum	2	0	0	1	0	0	0
gerd	730	minimum	2	0	0.01	0.99	0	0	0
gerd	30	mean	2	0	0	1	0	0	0
gerd_drugs	1095	latest	2	0	0.15	0.85	0	0	0
gerd_drugs	30	mean	2	0	0.02	0.98	0	0	0
gerd_drugs	365	maximum	2	0	0.09	0.91	0	0	0
gerd_drugs	1825	maximum	2	0	0.18	0.82	0	0	0
gerd_drugs	1460	maximum	2	0	0.17	0.83	0	0	0
gerd_drugs	730	maximum	2	0	0.13	0.87	0	0	0
gerd_drugs	1095	maximum	2	0	0.15	0.85	0	0	0
gerd_drugs	30	minimum	2	0	0.02	0.98	0	0	0
gerd_drugs	1825	latest	2	0	0.18	0.82	0	0	0
gerd_drugs	30	maximum	2	0	0.02	0.98	0	0	0
gerd_drugs	730	mean	2	0	0.13	0.87	0	0	0
gerd_drugs	180	minimum	2	0	0.06	0.94	0	0	0
gerd_drugs	365	minimum	2	0	0.09	0.91	0	0	0
gerd_drugs	730	minimum	2	0	0.13	0.87	0	0	0
gerd_drugs	180	maximum	2	0	0.06	0.94	0	0	0
gerd_drugs	1095	minimum	2	0	0.15	0.85	0	0	0
gerd_drugs	1825	minimum	2	0	0.18	0.82	0	0	0
gerd_drugs	180	mean	2	0	0.06	0.94	0	0	0
gerd_drugs	1460	latest	2	0	0.17	0.83	0	0	0
gerd_drugs	365	mean	2	0	0.09	0.91	0	0	0
gerd_drugs	1095	mean	2	0	0.15	0.85	0	0	0
gerd_drugs	1460	mean	2	0	0.17	0.83	0	0	0
gerd_drugs	1825	mean	2	0	0.18	0.82	0	0	0

gerd_drugs	30	latest	2	0	0.02	0.98	0	0	0
gerd_drugs	180	latest	2	0	0.06	0.94	0	0	0
gerd_drugs	365	latest	2	0	0.09	0.91	0	0	0
gerd_drugs	730	latest	2	0	0.13	0.87	0	0	0
gerd_drugs	1460	minimum	2	0	0.17	0.83	0	0	0
hba1c	180	maximum	57	nan	34.18	0.53	32	34	37
hba1c	30	maximum	53	nan	33.99	0.89	32	34	36
hba1c	365	maximum	56	nan	34.36	0.34	32	34	37
hba1c	1095	minimum	61	nan	32.73	0.18	31	33	35
hba1c	1825	minimum	64	nan	32.46	0.16	30	33	35
hba1c	1095	maximum	48	nan	34.91	0.18	32	35	37
hba1c	30	latest	54	nan	33.96	0.89	31	34	36
hba1c	365	latest	62	nan	33.9	0.34	31	34	36
hba1c	30	mean	135	nan	33.97	0.89	31	34	36
hba1c	1460	minimum	64	nan	32.57	0.17	30	33	35
hba1c	1825	mean	2829	nan	33.83	0.16	31.6	33.8	36
hba1c	1460	mean	2461	nan	33.83	0.17	31.6	33.8	36
hba1c	1095	mean	2003	nan	33.84	0.18	31.5	34	36
hba1c	730	mean	1437	nan	33.85	0.22	31.5	34	36
hba1c	180	mean	456	nan	33.95	0.53	31.5	34	36
hba1c	365	mean	785	nan	33.9	0.34	31.5	34	36
hba1c	180	latest	60	nan	33.95	0.53	31	34	36
hba1c	1825	latest	65	nan	33.84	0.16	31	34	36
hba1c	1460	maximum	47	nan	35.07	0.17	33	35	37
hba1c	730	minimum	60	nan	33	0.22	31	33	35
hba1c	365	minimum	62	nan	33.43	0.34	31	33	36
hba1c	180	minimum	60	nan	33.73	0.53	31	34	36
hba1c	30	minimum	54	nan	33.94	0.89	31	34	36

hba1c	1825	maximum	45	nan	35.16	0.16	33	35	38
hba1c	1460	latest	65	nan	33.84	0.17	31	34	36
hba1c	1095	latest	64	nan	33.85	0.18	31	34	36
hba1c	730	latest	63	nan	33.86	0.22	31	34	36
hba1c	730	maximum	51	nan	34.68	0.22	32	35	37
hdl	730	maximum	273	nan	1.47	0.23	1.2	1.4	1.7
hdl	30	maximum	234	nan	1.37	0.89	1.1	1.3	1.6
hdl	365	maximum	265	nan	1.43	0.34	1.1	1.4	1.7
hdl	180	maximum	257	nan	1.4	0.53	1.1	1.3	1.6
hdl	1095	latest	278	nan	1.38	0.19	1.1	1.3	1.6
hdl	1460	latest	279	nan	1.38	0.17	1.1	1.3	1.6
hdl	30	latest	234	nan	1.37	0.89	1.1	1.3	1.6
hdl	365	latest	270	nan	1.37	0.34	1.1	1.3	1.6
hdl	1095	minimum	258	nan	1.28	0.19	1	1.2	1.5
hdl	730	latest	275	nan	1.38	0.23	1.1	1.3	1.6
hdl	1825	minimum	261	nan	1.26	0.16	1	1.2	1.5
hdl	30	mean	440	nan	1.37	0.89	1.1	1.3	1.6
hdl	180	mean	1415	nan	1.37	0.53	1.1	1.3	1.6
hdl	1460	minimum	257	nan	1.27	0.17	1	1.2	1.5
hdl	730	mean	4382	nan	1.38	0.23	1.1	1.3	1.6
hdl	1460	maximum	280	nan	1.52	0.17	1.2	1.4	1.8
hdl	1095	mean	6109	nan	1.39	0.19	1.1	1.3	1.6
hdl	1825	mean	8513	nan	1.39	0.16	1.1	1.3	1.6
hdl	180	latest	262	nan	1.37	0.53	1.1	1.3	1.6
hdl	365	mean	2451	nan	1.38	0.34	1.1	1.3	1.6
hdl	730	minimum	262	nan	1.3	0.23	1	1.2	1.5
hdl	365	minimum	265	nan	1.33	0.34	1	1.3	1.5
hdl	1095	maximum	280	nan	1.5	0.19	1.2	1.4	1.7

hdl	30	minimum	233	nan	1.37	0.89	1.1	1.3	1.6
hdl	1825	maximum	284	nan	1.53	0.16	1.2	1.5	1.8
hdl	180	minimum	259	nan	1.35	0.53	1.1	1.3	1.6
hdl	1460	mean	7421	nan	1.39	0.17	1.1	1.3	1.6
hdl	1825	latest	280	nan	1.38	0.16	1.1	1.3	1.6
height_in_cm	730	latest	785	nan	170.45	0.27	165	171	178
height_in_cm	1095	latest	798	nan	170.45	0.22	165	171	178
height_in_cm	1460	latest	803	nan	170.47	0.2	165	171	178
height_in_cm	365	latest	764	nan	170.49	0.39	165	171	178
height_in_cm	180	latest	719	nan	170.44	0.55	165	171	178
height_in_cm	30	latest	550	nan	169.86	0.86	165	170	177
height_in_cm	1825	latest	808	nan	170.49	0.18	165	171	178
hyperlipidemia	365	maximum	2	0	0	1	0	0	0
hyperlipidemia	180	maximum	2	0	0	1	0	0	0
hyperlipidemia	30	maximum	2	0	0	1	0	0	0
hyperlipidemia	730	minimum	2	0	0	1	0	0	0
hyperlipidemia	30	latest	2	0	0	1	0	0	0
hyperlipidemia	365	minimum	2	0	0	1	0	0	0
hyperlipidemia	180	latest	2	0	0	1	0	0	0
hyperlipidemia	730	maximum	2	0	0	1	0	0	0
hyperlipidemia	730	latest	2	0	0	1	0	0	0
hyperlipidemia	1095	latest	2	0	0	1	0	0	0
hyperlipidemia	1460	latest	2	0	0	1	0	0	0
hyperlipidemia	1825	latest	2	0	0	1	0	0	0
hyperlipidemia	365	latest	2	0	0	1	0	0	0
hyperlipidemia	1825	mean	2	0	0	1	0	0	0
hyperlipidemia	365	mean	2	0	0	1	0	0	0
hyperlipidemia	1825	maximum	2	0	0	1	0	0	0

hyperlipidemia	1460	maximum	2	0	0	1	0	0	0
hyperlipidemia	1460	mean	2	0	0	1	0	0	0
hyperlipidemia	1095	minimum	2	0	0	1	0	0	0
hyperlipidemia	1095	mean	2	0	0	1	0	0	0
hyperlipidemia	180	mean	2	0	0	1	0	0	0
hyperlipidemia	730	mean	2	0	0	1	0	0	0
hyperlipidemia	1825	minimum	2	0	0	1	0	0	0
hyperlipidemia	1460	minimum	2	0	0	1	0	0	0
hyperlipidemia	1095	maximum	2	0	0	1	0	0	0
hyperlipidemia	180	minimum	2	0	0	1	0	0	0
hyperlipidemia	30	minimum	2	0	0	1	0	0	0
hyperlipidemia	30	mean	2	0	0	1	0	0	0
lamotrigine	180	latest	2	0	0.03	0.97	0	0	0
lamotrigine	1095	mean	2	0	0.06	0.94	0	0	0
lamotrigine	1825	mean	2	0	0.07	0.93	0	0	0
lamotrigine	30	latest	2	0	0.01	0.99	0	0	0
lamotrigine	365	latest	2	0	0.04	0.96	0	0	0
lamotrigine	730	mean	2	0	0.05	0.95	0	0	0
lamotrigine	1095	latest	2	0	0.06	0.94	0	0	0
lamotrigine	1460	latest	2	0	0.07	0.93	0	0	0
lamotrigine	1825	latest	2	0	0.07	0.93	0	0	0
lamotrigine	30	maximum	2	0	0.01	0.99	0	0	0
lamotrigine	180	maximum	2	0	0.03	0.97	0	0	0
lamotrigine	180	mean	2	0	0.03	0.97	0	0	0
lamotrigine	730	latest	2	0	0.05	0.95	0	0	0
lamotrigine	1460	mean	2	0	0.07	0.93	0	0	0
lamotrigine	365	maximum	2	0	0.04	0.96	0	0	0
lamotrigine	1460	maximum	2	0	0.07	0.93	0	0	0

lamotrigine	30	mean	2	0	0.01	0.99	0	0	0
lamotrigine	1825	minimum	2	0	0.07	0.93	0	0	0
lamotrigine	1825	maximum	2	0	0.07	0.93	0	0	0
lamotrigine	1095	minimum	2	0	0.06	0.94	0	0	0
lamotrigine	1460	minimum	2	0	0.07	0.93	0	0	0
lamotrigine	1095	maximum	2	0	0.06	0.94	0	0	0
lamotrigine	730	minimum	2	0	0.05	0.95	0	0	0
lamotrigine	365	minimum	2	0	0.04	0.96	0	0	0
lamotrigine	365	mean	2	0	0.04	0.96	0	0	0
lamotrigine	180	minimum	2	0	0.03	0.97	0	0	0
lamotrigine	30	minimum	2	0	0.01	0.99	0	0	0
lamotrigine	730	maximum	2	0	0.05	0.95	0	0	0
ldl	1095	latest	87	nan	2.84	0.24	2.2	2.8	3.4
ldl	1460	latest	87	nan	2.84	0.22	2.2	2.8	3.4
ldl	1825	latest	87	nan	2.84	0.21	2.2	2.8	3.4
ldl	1825	minimum	79	nan	2.53	0.21	1.9	2.5	3
ldl	30	maximum	82	nan	2.87	0.9	2.2	2.8	3.4
ldl	180	maximum	86	nan	2.93	0.57	2.3	2.8	3.5
ldl	1460	maximum	95	nan	3.18	0.22	2.5	3.1	3.8
ldl	1095	maximum	94	nan	3.14	0.24	2.4	3	3.7
ldl	1825	maximum	93	nan	3.2	0.21	2.5	3.1	3.8
ldl	180	minimum	84	nan	2.8	0.57	2.2	2.7	3.4
ldl	1460	minimum	82	nan	2.55	0.22	2	2.5	3.1
ldl	730	latest	87	nan	2.84	0.28	2.2	2.8	3.4
ldl	730	maximum	93	nan	3.08	0.28	2.4	3	3.7
ldl	365	latest	87	nan	2.85	0.4	2.2	2.8	3.4
ldl	365	maximum	91	nan	2.99	0.4	2.3	2.9	3.6
ldl	30	latest	82	nan	2.86	0.9	2.2	2.8	3.4

ldl	730	minimum	86	nan	2.64	0.28	2	2.6	3.2
ldl	1095	minimum	85	nan	2.58	0.24	2	2.5	3.1
ldl	365	minimum	87	nan	2.73	0.4	2.1	2.7	3.3
ldl	30	minimum	83	nan	2.86	0.9	2.2	2.8	3.4
ldl	30	mean	247	nan	2.86	0.9	2.2	2.8	3.4
ldl	180	latest	85	nan	2.86	0.57	2.2	2.8	3.4
ldl	365	mean	1403	nan	2.86	0.4	2.2	2.8	3.4
ldl	730	mean	2329	nan	2.86	0.28	2.2	2.8	3.4
ldl	1095	mean	3124	nan	2.86	0.24	2.2	2.8	3.4
ldl	1460	mean	3751	nan	2.86	0.22	2.3	2.8	3.4
ldl	1825	mean	4228	nan	2.86	0.21	2.3	2.8	3.4
ldl	180	mean	798	nan	2.87	0.57	2.2	2.8	3.4
lithium	180	minimum	2	0	0.02	0.98	0	0	0
lithium	1460	minimum	2	0	0.04	0.96	0	0	0
lithium	1095	minimum	2	0	0.03	0.97	0	0	0
lithium	730	minimum	2	0	0.03	0.97	0	0	0
lithium	365	minimum	2	0	0.02	0.98	0	0	0
lithium	30	minimum	2	0	0.01	0.99	0	0	0
lithium	180	maximum	2	0	0.02	0.98	0	0	0
lithium	1460	maximum	2	0	0.04	0.96	0	0	0
lithium	1095	maximum	2	0	0.03	0.97	0	0	0
lithium	730	maximum	2	0	0.03	0.97	0	0	0
lithium	30	mean	2	0	0.01	0.99	0	0	0
lithium	1825	minimum	2	0	0.04	0.96	0	0	0
lithium	1825	maximum	2	0	0.04	0.96	0	0	0
lithium	180	mean	2	0	0.02	0.98	0	0	0
lithium	30	maximum	2	0	0.01	0.99	0	0	0
lithium	365	mean	2	0	0.02	0.98	0	0	0

lithium	365	maximum	2	0	0.02	0.98	0	0	0
lithium	1825	latest	2	0	0.04	0.96	0	0	0
lithium	1460	latest	2	0	0.04	0.96	0	0	0
lithium	1095	latest	2	0	0.03	0.97	0	0	0
lithium	730	latest	2	0	0.03	0.97	0	0	0
lithium	1825	mean	2	0	0.04	0.96	0	0	0
lithium	180	latest	2	0	0.02	0.98	0	0	0
lithium	30	latest	2	0	0.01	0.99	0	0	0
lithium	1460	mean	2	0	0.04	0.96	0	0	0
lithium	1095	mean	2	0	0.03	0.97	0	0	0
lithium	730	mean	2	0	0.03	0.97	0	0	0
lithium	365	latest	2	0	0.02	0.98	0	0	0
ogtt	1825	minimum	78	nan	6.54	0.99	5.6	6.4	7.4
ogtt	730	latest	76	nan	6.86	0.99	5.8	6.7	7.8
ogtt	365	latest	70	nan	6.79	1	5.8	6.7	7.8
ogtt	180	latest	70	nan	6.72	1	5.8	6.7	7.8
ogtt	1095	latest	78	nan	6.88	0.99	5.9	6.7	7.8
ogtt	1460	mean	167	nan	6.78	0.99	5.8	6.7	7.7
ogtt	30	latest	54	nan	6.78	1	5.9	6.6	7.8
ogtt	1095	mean	159	nan	6.84	0.99	5.9	6.7	7.8
ogtt	730	mean	138	nan	6.81	0.99	5.8	6.7	7.8
ogtt	365	mean	108	nan	6.74	1	5.8	6.7	7.8
ogtt	30	mean	56	nan	6.79	1	5.9	6.6	7.8
ogtt	1825	mean	177	nan	6.74	0.99	5.8	6.6	7.6
ogtt	1460	maximum	77	nan	6.96	0.99	5.9	6.8	7.9
ogtt	180	mean	96	nan	6.68	1	5.8	6.6	7.7
ogtt	1095	minimum	78	nan	6.68	0.99	5.7	6.6	7.6
ogtt	365	minimum	69	nan	6.64	1	5.7	6.6	7.6

ogtt	1460	latest	78	nan	6.81	0.99	5.8	6.7	7.7
ogtt	1825	latest	78	nan	6.76	0.99	5.7	6.6	7.7
ogtt	30	maximum	54	nan	6.79	1	5.9	6.6	7.8
ogtt	180	maximum	70	nan	6.75	1	5.8	6.7	7.8
ogtt	365	maximum	70	nan	6.84	1	5.9	6.7	7.8
ogtt	1460	minimum	78	nan	6.59	0.99	5.6	6.5	7.5
ogtt	1095	maximum	77	nan	7.01	0.99	5.9	6.8	7.9
ogtt	1825	maximum	77	nan	6.94	0.99	5.9	6.8	7.8
ogtt	30	minimum	54	nan	6.78	1	5.9	6.6	7.8
ogtt	180	minimum	69	nan	6.61	1	5.7	6.5	7.6
ogtt	730	minimum	76	nan	6.7	0.99	5.7	6.6	7.7
ogtt	730	maximum	76	nan	6.93	0.99	5.8	6.7	7.9
polycystic_ovarian_syndrome	1095	maximum	2	0	0	1	0	0	0
polycystic_ovarian_syndrome	180	latest	2	0	0	1	0	0	0
polycystic_ovarian_syndrome	365	maximum	2	0	0	1	0	0	0
polycystic_ovarian_syndrome	180	maximum	2	0	0	1	0	0	0
polycystic_ovarian_syndrome	30	maximum	2	0	0	1	0	0	0
polycystic_ovarian_syndrome	1095	latest	2	0	0	1	0	0	0
polycystic_ovarian_syndrome	730	latest	2	0	0	1	0	0	0
polycystic_ovarian_syndrome	1825	minimum	2	0	0.01	0.99	0	0	0
polycystic_ovarian_syndrome	1460	latest	2	0	0	1	0	0	0
polycystic_ovarian_syndrome	1825	latest	2	0	0.01	0.99	0	0	0
polycystic_ovarian_syndrome	730	maximum	2	0	0	1	0	0	0
polycystic_ovarian_syndrome	365	latest	2	0	0	1	0	0	0
polycystic_ovarian_syndrome	1825	mean	2	0	0.01	0.99	0	0	0
polycystic_ovarian_syndrome	30	latest	2	0	0	1	0	0	0
polycystic_ovarian_syndrome	1460	mean	2	0	0	1	0	0	0
polycystic_ovarian_syndrome	1825	maximum	2	0	0.01	0.99	0	0	0

polycystic_ovarian_syndrome	180	minimum	2	0	0	1	0	0	0
polycystic_ovarian_syndrome	365	minimum	2	0	0	1	0	0	0
polycystic_ovarian_syndrome	730	minimum	2	0	0	1	0	0	0
polycystic_ovarian_syndrome	1095	minimum	2	0	0	1	0	0	0
polycystic_ovarian_syndrome	30	minimum	2	0	0	1	0	0	0
polycystic_ovarian_syndrome	1460	maximum	2	0	0	1	0	0	0
polycystic_ovarian_syndrome	1095	mean	2	0	0	1	0	0	0
polycystic_ovarian_syndrome	30	mean	2	0	0	1	0	0	0
polycystic_ovarian_syndrome	180	mean	2	0	0	1	0	0	0
polycystic_ovarian_syndrome	365	mean	2	0	0	1	0	0	0
polycystic_ovarian_syndrome	730	mean	2	0	0	1	0	0	0
polycystic_ovarian_syndrome	1460	minimum	2	0	0	1	0	0	0
pregabaline	1095	mean	2	0	0.05	0.95	0	0	0
pregabaline	730	mean	2	0	0.04	0.96	0	0	0
pregabaline	365	mean	2	0	0.03	0.97	0	0	0
pregabaline	180	mean	2	0	0.02	0.98	0	0	0
pregabaline	30	mean	2	0	0.01	0.99	0	0	0
pregabaline	1825	minimum	2	0	0.06	0.94	0	0	0
pregabaline	730	minimum	2	0	0.04	0.96	0	0	0
pregabaline	1095	minimum	2	0	0.05	0.95	0	0	0
pregabaline	180	maximum	2	0	0.02	0.98	0	0	0
pregabaline	30	maximum	2	0	0.01	0.99	0	0	0
pregabaline	365	minimum	2	0	0.03	0.97	0	0	0
pregabaline	1460	mean	2	0	0.06	0.94	0	0	0
pregabaline	1460	minimum	2	0	0.06	0.94	0	0	0
pregabaline	180	minimum	2	0	0.02	0.98	0	0	0
pregabaline	1825	mean	2	0	0.06	0.94	0	0	0
pregabaline	180	latest	2	0	0.02	0.98	0	0	0

pregabaline	30	latest	2	0	0.01	0.99	0	0	0
pregabaline	365	maximum	2	0	0.03	0.97	0	0	0
pregabaline	730	maximum	2	0	0.04	0.96	0	0	0
pregabaline	1095	maximum	2	0	0.05	0.95	0	0	0
pregabaline	1825	maximum	2	0	0.06	0.94	0	0	0
pregabaline	1460	latest	2	0	0.06	0.94	0	0	0
pregabaline	1095	latest	2	0	0.05	0.95	0	0	0
pregabaline	1460	maximum	2	0	0.06	0.94	0	0	0
pregabaline	30	minimum	2	0	0.01	0.99	0	0	0
pregabaline	730	latest	2	0	0.04	0.96	0	0	0
pregabaline	365	latest	2	0	0.03	0.97	0	0	0
pregabaline	1825	latest	2	0	0.06	0.94	0	0	0
scheduled_glc	180	latest	106	nan	6.06	0.99	5.1	5.7	6.6
scheduled_glc	365	latest	114	nan	5.99	0.98	5.1	5.6	6.5
scheduled_glc	730	latest	119	nan	5.94	0.96	5	5.6	6.4
scheduled_glc	1095	latest	121	nan	5.92	0.95	5	5.6	6.4
scheduled_glc	1460	latest	122	nan	5.92	0.94	5	5.6	6.5
scheduled_glc	365	maximum	156	nan	6.51	0.98	5.2	5.8	7.1
scheduled_glc	180	maximum	138	nan	6.57	0.99	5.2	5.9	7.2
scheduled_glc	1825	minimum	116	nan	5.52	0.93	4.8	5.3	6.1
scheduled_glc	1095	maximum	166	nan	6.44	0.95	5.2	5.8	7.1
scheduled_glc	1460	maximum	167	nan	6.43	0.94	5.2	5.8	7.1
scheduled_glc	730	maximum	163	nan	6.45	0.96	5.2	5.8	7.1
scheduled_glc	1825	latest	122	nan	5.92	0.93	5	5.6	6.5
scheduled_glc	30	latest	95	nan	6.11	1	5.1	5.7	6.7
scheduled_glc	1825	mean	927	nan	5.88	0.93	5.1	5.6	6.4
scheduled_glc	1095	mean	892	nan	5.89	0.95	5.1	5.6	6.4
scheduled_glc	30	maximum	115	nan	6.54	1	5.2	5.9	7.2

scheduled_glc	1825	maximum	167	nan	6.42	0.93	5.2	5.8	7.1
scheduled_glc	30	minimum	99	nan	5.86	1	4.9	5.6	6.4
scheduled_glc	180	minimum	110	nan	5.76	0.99	4.9	5.4	6.3
scheduled_glc	365	minimum	114	nan	5.63	0.98	4.8	5.4	6.2
scheduled_glc	730	minimum	116	nan	5.57	0.96	4.8	5.3	6.1
scheduled_glc	1460	mean	910	nan	5.89	0.94	5.1	5.6	6.4
scheduled_glc	1460	minimum	115	nan	5.53	0.94	4.8	5.3	6.1
scheduled_glc	30	mean	322	nan	6.13	1	5.2	5.8	6.8
scheduled_glc	180	mean	592	nan	6.08	0.99	5.2	5.7	6.7
scheduled_glc	365	mean	717	nan	5.98	0.98	5.1	5.6	6.6
scheduled_glc	730	mean	834	nan	5.92	0.96	5.1	5.6	6.5
scheduled_glc	1095	minimum	115	nan	5.54	0.95	4.8	5.3	6.1
selected_nassa	1460	maximum	2	0	0.06	0.94	0	0	0
selected_nassa	1095	maximum	2	0	0.06	0.94	0	0	0
selected_nassa	730	maximum	2	0	0.05	0.95	0	0	0
selected_nassa	365	maximum	2	0	0.04	0.96	0	0	0
selected_nassa	180	maximum	2	0	0.02	0.98	0	0	0
selected_nassa	1460	latest	2	0	0.06	0.94	0	0	0
selected_nassa	1825	latest	2	0	0.07	0.93	0	0	0
selected_nassa	1095	latest	2	0	0.06	0.94	0	0	0
selected_nassa	730	latest	2	0	0.05	0.95	0	0	0
selected_nassa	365	latest	2	0	0.04	0.96	0	0	0
selected_nassa	1825	maximum	2	0	0.07	0.93	0	0	0
selected_nassa	30	maximum	2	0	0.01	0.99	0	0	0
selected_nassa	180	minimum	2	0	0.02	0.98	0	0	0
selected_nassa	180	mean	2	0	0.02	0.98	0	0	0
selected_nassa	730	minimum	2	0	0.05	0.95	0	0	0
selected_nassa	1095	minimum	2	0	0.06	0.94	0	0	0

selected_nassa	1460	minimum	2	0	0.06	0.94	0	0	0
selected_nassa	1825	minimum	2	0	0.07	0.93	0	0	0
selected_nassa	30	mean	2	0	0.01	0.99	0	0	0
selected_nassa	30	minimum	2	0	0.01	0.99	0	0	0
selected_nassa	365	mean	2	0	0.04	0.96	0	0	0
selected_nassa	730	mean	2	0	0.05	0.95	0	0	0
selected_nassa	1095	mean	2	0	0.06	0.94	0	0	0
selected_nassa	1460	mean	2	0	0.06	0.94	0	0	0
selected_nassa	1825	mean	2	0	0.07	0.93	0	0	0
selected_nassa	30	latest	2	0	0.01	0.99	0	0	0
selected_nassa	365	minimum	2	0	0.04	0.96	0	0	0
selected_nassa	180	latest	2	0	0.02	0.98	0	0	0
sex_female	nan	nan	2	nan	0.62	nan	nan	nan	nan
sleep_apnea	1460	latest	2	0	0.01	0.99	0	0	0
sleep_apnea	1825	mean	2	0	0.01	0.99	0	0	0
sleep_apnea	180	mean	2	0	0	1	0	0	0
sleep_apnea	365	mean	2	0	0.01	0.99	0	0	0
sleep_apnea	730	mean	2	0	0.01	0.99	0	0	0
sleep_apnea	1095	mean	2	0	0.01	0.99	0	0	0
sleep_apnea	1460	mean	2	0	0.01	0.99	0	0	0
sleep_apnea	1095	latest	2	0	0.01	0.99	0	0	0
sleep_apnea	180	latest	2	0	0	1	0	0	0
sleep_apnea	365	latest	2	0	0.01	0.99	0	0	0
sleep_apnea	730	latest	2	0	0.01	0.99	0	0	0
sleep_apnea	1825	minimum	2	0	0.01	0.99	0	0	0
sleep_apnea	30	mean	2	0	0	1	0	0	0
sleep_apnea	30	maximum	2	0	0	1	0	0	0
sleep_apnea	30	latest	2	0	0	1	0	0	0

sleep_apnea	1095	minimum	2	0	0.01	0.99	0	0	0
sleep_apnea	1460	minimum	2	0	0.01	0.99	0	0	0
sleep_apnea	1825	latest	2	0	0.01	0.99	0	0	0
sleep_apnea	180	maximum	2	0	0	1	0	0	0
sleep_apnea	365	maximum	2	0	0.01	0.99	0	0	0
sleep_apnea	1095	maximum	2	0	0.01	0.99	0	0	0
sleep_apnea	1460	maximum	2	0	0.01	0.99	0	0	0
sleep_apnea	730	maximum	2	0	0.01	0.99	0	0	0
sleep_apnea	30	minimum	2	0	0	1	0	0	0
sleep_apnea	180	minimum	2	0	0	1	0	0	0
sleep_apnea	365	minimum	2	0	0.01	0.99	0	0	0
sleep_apnea	730	minimum	2	0	0.01	0.99	0	0	0
sleep_apnea	1825	maximum	2	0	0.01	0.99	0	0	0
snri	1095	mean	2	0	0.09	0.91	0	0	0
snri	365	latest	2	0	0.06	0.94	0	0	0
snri	1825	mean	2	0	0.11	0.89	0	0	0
snri	30	latest	2	0	0.01	0.99	0	0	0
snri	180	latest	2	0	0.04	0.96	0	0	0
snri	730	latest	2	0	0.08	0.92	0	0	0
snri	1460	latest	2	0	0.1	0.9	0	0	0
snri	180	maximum	2	0	0.04	0.96	0	0	0
snri	30	mean	2	0	0.01	0.99	0	0	0
snri	30	maximum	2	0	0.01	0.99	0	0	0
snri	1825	latest	2	0	0.11	0.89	0	0	0
snri	730	mean	2	0	0.08	0.92	0	0	0
snri	1095	latest	2	0	0.09	0.91	0	0	0
snri	365	mean	2	0	0.06	0.94	0	0	0
snri	180	mean	2	0	0.04	0.96	0	0	0

snri	1460	mean	2	0	0.1	0.9	0	0	0
snri	730	minimum	2	0	0.08	0.92	0	0	0
snri	365	minimum	2	0	0.06	0.94	0	0	0
snri	180	minimum	2	0	0.04	0.96	0	0	0
snri	1460	maximum	2	0	0.1	0.9	0	0	0
snri	1825	maximum	2	0	0.11	0.89	0	0	0
snri	1095	minimum	2	0	0.09	0.91	0	0	0
snri	730	maximum	2	0	0.08	0.92	0	0	0
snri	1460	minimum	2	0	0.1	0.9	0	0	0
snri	365	maximum	2	0	0.06	0.94	0	0	0
snri	30	minimum	2	0	0.01	0.99	0	0	0
snri	1825	minimum	2	0	0.11	0.89	0	0	0
snri	1095	maximum	2	0	0.09	0.91	0	0	0
ssri	730	mean	2	0	0.12	0.88	0	0	0
ssri	1095	mean	2	0	0.15	0.85	0	0	0
ssri	1460	mean	2	0	0.16	0.84	0	0	0
ssri	1825	mean	2	0	0.17	0.83	0	0	0
ssri	30	latest	2	0	0.02	0.98	0	0	0
ssri	1095	latest	2	0	0.15	0.85	0	0	0
ssri	365	latest	2	0	0.09	0.91	0	0	0
ssri	1825	latest	2	0	0.17	0.83	0	0	0
ssri	1825	minimum	2	0	0.17	0.83	0	0	0
ssri	730	latest	2	0	0.12	0.88	0	0	0
ssri	365	mean	2	0	0.09	0.91	0	0	0
ssri	180	latest	2	0	0.06	0.94	0	0	0
ssri	180	mean	2	0	0.06	0.94	0	0	0
ssri	30	mean	2	0	0.02	0.98	0	0	0
ssri	1460	latest	2	0	0.16	0.84	0	0	0

ssri	1460	minimum	2	0	0.16	0.84	0	0	0
ssri	30	maximum	2	0	0.02	0.98	0	0	0
ssri	180	maximum	2	0	0.06	0.94	0	0	0
ssri	365	maximum	2	0	0.09	0.91	0	0	0
ssri	1095	maximum	2	0	0.15	0.85	0	0	0
ssri	1460	maximum	2	0	0.16	0.84	0	0	0
ssri	730	maximum	2	0	0.12	0.88	0	0	0
ssri	30	minimum	2	0	0.02	0.98	0	0	0
ssri	180	minimum	2	0	0.06	0.94	0	0	0
ssri	365	minimum	2	0	0.09	0.91	0	0	0
ssri	730	minimum	2	0	0.12	0.88	0	0	0
ssri	1095	minimum	2	0	0.15	0.85	0	0	0
ssri	1825	maximum	2	0	0.17	0.83	0	0	0
statins	180	latest	2	0	0.02	0.98	0	0	0
statins	1095	mean	2	0	0.03	0.97	0	0	0
statins	1460	mean	2	0	0.03	0.97	0	0	0
statins	1825	mean	2	0	0.03	0.97	0	0	0
statins	30	latest	2	0	0.01	0.99	0	0	0
statins	365	latest	2	0	0.02	0.98	0	0	0
statins	730	mean	2	0	0.03	0.97	0	0	0
statins	1095	latest	2	0	0.03	0.97	0	0	0
statins	1460	latest	2	0	0.03	0.97	0	0	0
statins	1825	latest	2	0	0.03	0.97	0	0	0
statins	365	mean	2	0	0.02	0.98	0	0	0
statins	180	maximum	2	0	0.02	0.98	0	0	0
statins	730	latest	2	0	0.03	0.97	0	0	0
statins	180	mean	2	0	0.02	0.98	0	0	0
statins	1825	minimum	2	0	0.03	0.97	0	0	0

statins	30	mean	2	0	0.01	0.99	0	0	0
statins	30	maximum	2	0	0.01	0.99	0	0	0
statins	365	maximum	2	0	0.02	0.98	0	0	0
statins	730	maximum	2	0	0.03	0.97	0	0	0
statins	1460	maximum	2	0	0.03	0.97	0	0	0
statins	1825	maximum	2	0	0.03	0.97	0	0	0
statins	1095	maximum	2	0	0.03	0.97	0	0	0
statins	180	minimum	2	0	0.02	0.98	0	0	0
statins	365	minimum	2	0	0.02	0.98	0	0	0
statins	730	minimum	2	0	0.03	0.97	0	0	0
statins	1095	minimum	2	0	0.03	0.97	0	0	0
statins	1460	minimum	2	0	0.03	0.97	0	0	0
statins	30	minimum	2	0	0.01	0.99	0	0	0
tca	30	mean	2	0	0.01	0.99	0	0	0
tca	180	mean	2	0	0.02	0.98	0	0	0
tca	1095	latest	2	0	0.05	0.95	0	0	0
tca	1460	mean	2	0	0.05	0.95	0	0	0
tca	730	latest	2	0	0.04	0.96	0	0	0
tca	180	latest	2	0	0.02	0.98	0	0	0
tca	365	mean	2	0	0.03	0.97	0	0	0
tca	1825	mean	2	0	0.05	0.95	0	0	0
tca	365	latest	2	0	0.03	0.97	0	0	0
tca	1095	mean	2	0	0.05	0.95	0	0	0
tca	730	mean	2	0	0.04	0.96	0	0	0
tca	365	minimum	2	0	0.03	0.97	0	0	0
tca	30	latest	2	0	0.01	0.99	0	0	0
tca	1825	minimum	2	0	0.05	0.95	0	0	0
tca	30	minimum	2	0	0.01	0.99	0	0	0

tca	1095	minimum	2	0	0.05	0.95	0	0	0
tca	30	maximum	2	0	0.01	0.99	0	0	0
tca	180	maximum	2	0	0.02	0.98	0	0	0
tca	1460	latest	2	0	0.05	0.95	0	0	0
tca	365	maximum	2	0	0.03	0.97	0	0	0
tca	730	maximum	2	0	0.04	0.96	0	0	0
tca	1460	minimum	2	0	0.05	0.95	0	0	0
tca	1095	maximum	2	0	0.05	0.95	0	0	0
tca	1460	maximum	2	0	0.05	0.95	0	0	0
tca	1825	maximum	2	0	0.05	0.95	0	0	0
tca	180	minimum	2	0	0.02	0.98	0	0	0
tca	730	minimum	2	0	0.04	0.96	0	0	0
tca	1825	latest	2	0	0.05	0.95	0	0	0
top_10_weight_gaining_antipsychotics	1825	maximum	2	0	0.28	0.72	0	0	1
top_10_weight_gaining_antipsychotics	1460	maximum	2	0	0.28	0.72	0	0	1
top_10_weight_gaining_antipsychotics	1095	maximum	2	0	0.26	0.74	0	0	1
top_10_weight_gaining_antipsychotics	730	maximum	2	0	0.24	0.76	0	0	0
top_10_weight_gaining_antipsychotics	365	maximum	2	0	0.19	0.81	0	0	0
top_10_weight_gaining_antipsychotics	1460	minimum	2	0	0.28	0.72	0	0	1
top_10_weight_gaining_antipsychotics	1095	latest	2	0	0.26	0.74	0	0	1
top_10_weight_gaining_antipsychotics	1460	latest	2	0	0.28	0.72	0	0	1

top_10_weight_gaining_antipsychotics	180	minimum	2	0	0.15	0.85	0	0	0
top_10_weight_gaining_antipsychotics	1825	latest	2	0	0.28	0.72	0	0	1
top_10_weight_gaining_antipsychotics	30	minimum	2	0	0.06	0.94	0	0	0
top_10_weight_gaining_antipsychotics	30	maximum	2	0	0.06	0.94	0	0	0
top_10_weight_gaining_antipsychotics	365	minimum	2	0	0.19	0.81	0	0	0
top_10_weight_gaining_antipsychotics	730	minimum	2	0	0.24	0.76	0	0	0
top_10_weight_gaining_antipsychotics	30	mean	2	0	0.06	0.94	0	0	0
top_10_weight_gaining_antipsychotics	1095	minimum	2	0	0.26	0.74	0	0	1
top_10_weight_gaining_antipsychotics	365	latest	2	0	0.19	0.81	0	0	0
top_10_weight_gaining_antipsychotics	180	latest	2	0	0.15	0.85	0	0	0
top_10_weight_gaining_antipsychotics	30	latest	2	0	0.06	0.94	0	0	0
top_10_weight_gaining_antipsychotics	730	latest	2	0	0.24	0.76	0	0	0
top_10_weight_gaining_antipsychotics	1460	mean	2	0	0.28	0.72	0	0	1
top_10_weight_gaining_antipsychotics	1825	mean	2	0	0.28	0.72	0	0	1
top_10_weight_gaining_antipsychotics	730	mean	2	0	0.24	0.76	0	0	0
top_10_weight_gaining_antipsychotics	365	mean	2	0	0.19	0.81	0	0	0

top_10_weight_gaining_antipsychotics	180	mean	2	0	0.15	0.85	0	0	0
top_10_weight_gaining_antipsychotics	1825	minimum	2	0	0.28	0.72	0	0	1
top_10_weight_gaining_antipsychotics	180	maximum	2	0	0.15	0.85	0	0	0
top_10_weight_gaining_antipsychotics	1095	mean	2	0	0.26	0.74	0	0	1
triglycerides	1460	maximum	650	nan	1.86	0.26	1	1.5	2.3
triglycerides	180	maximum	610	nan	1.63	0.6	0.9	1.3	2
triglycerides	365	maximum	615	nan	1.69	0.43	0.9	1.4	2
triglycerides	730	maximum	633	nan	1.77	0.32	1	1.4	2.1
triglycerides	1095	maximum	647	nan	1.83	0.28	1	1.5	2.2
triglycerides	30	maximum	556	nan	1.58	0.91	0.9	1.3	1.9
triglycerides	1460	minimum	504	nan	1.2	0.26	0.7	1	1.4
triglycerides	30	minimum	551	nan	1.56	0.91	0.9	1.3	1.9
triglycerides	180	minimum	577	nan	1.47	0.6	0.8	1.2	1.8
triglycerides	365	minimum	557	nan	1.4	0.43	0.8	1.1	1.7
triglycerides	730	minimum	536	nan	1.29	0.32	0.8	1.1	1.6
triglycerides	1825	maximum	662	nan	1.88	0.25	1	1.5	2.3
triglycerides	1825	latest	632	nan	1.51	0.25	0.9	1.2	1.8
triglycerides	1095	minimum	517	nan	1.23	0.28	0.7	1	1.5
triglycerides	1460	latest	629	nan	1.51	0.26	0.9	1.2	1.8
triglycerides	1825	minimum	509	nan	1.18	0.25	0.7	1	1.4
triglycerides	1460	mean	7251	nan	1.49	0.26	0.9	1.2	1.8
triglycerides	1095	latest	627	nan	1.51	0.28	0.9	1.2	1.8
triglycerides	730	latest	623	nan	1.52	0.32	0.9	1.2	1.8
triglycerides	180	latest	600	nan	1.55	0.6	0.9	1.3	1.9
triglycerides	30	latest	552	nan	1.57	0.91	0.9	1.3	1.9

triglycerides	365	latest	612	nan	1.53	0.43	0.9	1.2	1.9
triglycerides	1095	mean	6262	nan	1.5	0.28	0.9	1.2	1.8
triglycerides	730	mean	4834	nan	1.51	0.32	0.9	1.2	1.8
triglycerides	365	mean	3064	nan	1.53	0.43	0.9	1.3	1.8
triglycerides	180	mean	1944	nan	1.55	0.6	0.9	1.3	1.9
triglycerides	30	mean	760	nan	1.57	0.91	0.9	1.3	1.9
triglycerides	1825	mean	8123	nan	1.48	0.25	0.9	1.2	1.8
unscheduled_p_glc	180	latest	95	nan	5.61	0.67	5	5.4	6
unscheduled_p_glc	1095	latest	96	nan	5.61	0.37	5	5.4	6
unscheduled_p_glc	30	latest	95	nan	5.61	0.91	5	5.4	6
unscheduled_p_glc	730	latest	96	nan	5.61	0.42	5	5.4	6
unscheduled_p_glc	1825	minimum	98	nan	5.19	0.32	4.7	5.1	5.6
unscheduled_p_glc	365	latest	95	nan	5.61	0.54	5	5.4	6
unscheduled_p_glc	180	mean	2168	nan	5.62	0.67	5.1	5.5	6
unscheduled_p_glc	1460	mean	5526	nan	5.63	0.33	5.1	5.5	6
unscheduled_p_glc	1095	mean	5077	nan	5.63	0.37	5.1	5.5	6
unscheduled_p_glc	730	mean	4326	nan	5.63	0.42	5.1	5.5	6
unscheduled_p_glc	365	mean	3040	nan	5.62	0.54	5.1	5.5	6
unscheduled_p_glc	1460	latest	96	nan	5.61	0.33	5	5.5	6
unscheduled_p_glc	1825	mean	5717	nan	5.63	0.32	5.1	5.5	6
unscheduled_p_glc	1825	latest	96	nan	5.61	0.32	5	5.5	6
unscheduled_p_glc	1460	maximum	88	nan	6.16	0.33	5.3	5.9	6.8
unscheduled_p_glc	1460	minimum	97	nan	5.21	0.33	4.7	5.1	5.6
unscheduled_p_glc	30	mean	780	nan	5.61	0.91	5	5.5	6.1
unscheduled_p_glc	730	minimum	97	nan	5.3	0.42	4.8	5.2	5.7
unscheduled_p_glc	365	minimum	96	nan	5.38	0.54	4.9	5.3	5.8
unscheduled_p_glc	180	minimum	96	nan	5.45	0.67	4.9	5.3	5.9
unscheduled_p_glc	1825	maximum	88	nan	6.2	0.32	5.3	5.9	6.8

unscheduled_p_glc	30	minimum	96	nan	5.53	0.91	5	5.4	5.9
unscheduled_p_glc	1095	maximum	88	nan	6.11	0.37	5.3	5.8	6.7
unscheduled_p_glc	365	maximum	90	nan	5.91	0.54	5.2	5.7	6.4
unscheduled_p_glc	180	maximum	92	nan	5.82	0.67	5.1	5.6	6.3
unscheduled_p_glc	30	maximum	91	nan	5.7	0.91	5	5.5	6.2
unscheduled_p_glc	730	maximum	90	nan	6.03	0.42	5.2	5.7	6.6
unscheduled_p_glc	1095	minimum	97	nan	5.24	0.37	4.8	5.2	5.6
urinary_glc	1825	mean	96	nan	0.02	0.78	0	0	0
urinary_glc	365	mean	50	nan	0.02	0.9	0	0	0
urinary_glc	730	mean	70	nan	0.02	0.85	0	0	0
urinary_glc	1095	mean	83	nan	0.02	0.81	0	0	0
urinary_glc	30	latest	5	nan	0.03	0.98	0	0	0
urinary_glc	365	latest	5	nan	0.02	0.9	0	0	0
urinary_glc	730	latest	5	nan	0.02	0.85	0	0	0
urinary_glc	1095	latest	5	nan	0.02	0.81	0	0	0
urinary_glc	1825	latest	5	nan	0.02	0.78	0	0	0
urinary_glc	1825	minimum	5	nan	0.01	0.78	0	0	0
urinary_glc	180	mean	39	nan	0.02	0.94	0	0	0
urinary_glc	180	latest	5	nan	0.02	0.94	0	0	0
urinary_glc	30	mean	16	nan	0.03	0.98	0	0	0
urinary_glc	1460	latest	5	nan	0.02	0.79	0	0	0
urinary_glc	1460	minimum	5	nan	0.01	0.79	0	0	0
urinary_glc	1460	mean	95	nan	0.02	0.79	0	0	0
urinary_glc	730	minimum	5	nan	0.01	0.85	0	0	0
urinary_glc	365	minimum	5	nan	0.01	0.9	0	0	0
urinary_glc	180	minimum	4	nan	0.02	0.94	0	0	0
urinary_glc	1825	maximum	5	nan	0.04	0.78	0	0	0
urinary_glc	30	maximum	5	nan	0.03	0.98	0	0	0

urinary_glc	30	minimum	5	nan	0.02	0.98	0	0	0
urinary_glc	730	maximum	5	nan	0.04	0.85	0	0	0
urinary_glc	365	maximum	5	nan	0.03	0.9	0	0	0
urinary_glc	180	maximum	5	nan	0.03	0.94	0	0	0
urinary_glc	1095	minimum	5	nan	0.01	0.81	0	0	0
urinary_glc	1460	maximum	5	nan	0.04	0.79	0	0	0
urinary_glc	1095	maximum	5	nan	0.04	0.81	0	0	0
valproate	730	latest	2	0	0.02	0.98	0	0	0
valproate	180	latest	2	0	0.01	0.99	0	0	0
valproate	730	mean	2	0	0.02	0.98	0	0	0
valproate	1095	latest	2	0	0.03	0.97	0	0	0
valproate	365	latest	2	0	0.02	0.98	0	0	0
valproate	1460	mean	2	0	0.03	0.97	0	0	0
valproate	30	mean	2	0	0	1	0	0	0
valproate	180	mean	2	0	0.01	0.99	0	0	0
valproate	365	mean	2	0	0.02	0.98	0	0	0
valproate	1825	mean	2	0	0.03	0.97	0	0	0
valproate	1095	mean	2	0	0.03	0.97	0	0	0
valproate	1825	latest	2	0	0.03	0.97	0	0	0
valproate	30	latest	2	0	0	1	0	0	0
valproate	1825	minimum	2	0	0.03	0.97	0	0	0
valproate	365	minimum	2	0	0.02	0.98	0	0	0
valproate	1095	minimum	2	0	0.03	0.97	0	0	0
valproate	30	maximum	2	0	0	1	0	0	0
valproate	1460	latest	2	0	0.03	0.97	0	0	0
valproate	1460	minimum	2	0	0.03	0.97	0	0	0
valproate	365	maximum	2	0	0.02	0.98	0	0	0
valproate	730	maximum	2	0	0.02	0.98	0	0	0

valproate	180	maximum	2	0	0.01	0.99	0	0	0
valproate	1460	maximum	2	0	0.03	0.97	0	0	0
valproate	1825	maximum	2	0	0.03	0.97	0	0	0
valproate	30	minimum	2	0	0	1	0	0	0
valproate	180	minimum	2	0	0.01	0.99	0	0	0
valproate	730	minimum	2	0	0.02	0.98	0	0	0
valproate	1095	maximum	2	0	0.03	0.97	0	0	0
weight_in_kg	730	latest	2066	nan	78.93	0.25	63	76	91
weight_in_kg	365	latest	2052	nan	78.99	0.36	63	76	91
weight_in_kg	30	latest	1963	nan	75.46	0.83	58.5	71.9	88.9
weight_in_kg	180	latest	2047	nan	78.71	0.5	62.6	75.7	91
weight_in_kg	1825	latest	2077	nan	78.75	0.17	63	75.7	90.4
weight_in_kg	1460	latest	2074	nan	78.8	0.18	63	75.8	90.6
weight_in_kg	1095	latest	2071	nan	78.87	0.2	63	76	90.9

Aggregation function: How to aggregate multiple values within the lookbehind days.
Fallback strategy: Which value to write if no values exist within the lookbehind days.
Lookbehind days: How far before the prediction time to aggregate values of the predictor.
Predictor: The type of value used to generate the function.
Proportion using fallback: How large a proportion of the prediction times that have no values within the lookbehind days. See also Figure 1 panel F.
Unique values in predictor: After aggregation of all values within lookbehind days, how many unique aggregated values are in the result.

Abbreviations: ALAT: Alanine aminotransferase. p-Glc: Plasma glucose. BMI: Body mass index. CRP: C-reactive protein. EGFR: Estimated glomerular filtration rate. LDL: Low-density lipoprotein. GERD: Gastro-esophageal reflux disorder. HbA1c: Hemoglobin A1c. HDL: High-density lipoprotein. OGTT: Oral glucose-tolerance test. Glc: Glucose. NASSA: Noradrenergic and specific serotonergic antidepressants. SNRI: Serotonin and noradrenaline reuptake inhibitor. SSRI: Selective serotonin reuptake inhibitor. TCA: Tricyclic antidepressants.

Aggregation function: How to aggregate multiple values within the lookbehind days.
Fallback strategy: Which value to write if no values exist within the lookbehind days**.**
Lookbehind days: How far before the prediction time to aggregate values of the predictor.
Predictor: The type of value used to generate the function.
Proportion using fallback: How large a proportion of the prediction times that have no values within the lookbehind days. See also Figure 1 panel F.
Unique values in predictor: After aggregation of all values within lookbehind days, how many unique aggregated values are in the result.

Abbreviations: ALAT: Alanine aminotransferase. p-Glc: Plasma glucose. BMI: Body mass index. CRP: C-reactive protein. EGFR: Estimated glomerular filtration rate. LDL: Low-density lipoprotein. GERD: Gastro-esophageal reflux disorder. HbA1c: Hemoglobin A1c. HDL: High-density lipoprotein. OGTT: Oral glucose-tolerance test. Glc: Glucose. NASSA: Noradrenergic and specific serotonergic antidepressants. SNRI: Serotonin and noradrenaline reuptake inhibitor. SSRI: Selective serotonin reuptake inhibitor. TCA: Tricyclic antidepressants.

Supplementary Table 3. Hyperparameters for model selection. Parameters for the best performing model in bold.

**Preprocessing**	
Imputation method	Either most frequent value, **mean**, median or no imputation.
Scaling	**z-score-normalisation** or no scaling
Predictor selection	Chi-squared or **no feature selection**
Predictor selection percentile[a]	Between 1 and 90 **(N/A)**.
Lookbehind combination (days)[b]	One of either<br>• [30, 180, 365, 730, 1095, 1460, 1825]<br>• [30, 1095, 1825]<br>• **[30, 365, 730]**<br>• [30, 365]<br>• [365]
**Model hyperparameters**	
**XGBoost**	
N estimators	[100; 1200], **238**
Alpha	$[10^{-8}; 0.1]$, $\mathbf{2.25 \cdot 10^{-6}}$
Lambda	$[10^{-8}; 1.0]$, $\mathbf{8.6 \cdot 10^{-7}}$
Max depth	[1; 10], 2
Learning rate	$[10^{-8}; 1]$, **0.052**
Gamma	$[10^{-8}; 10^{-4}]$, **0.00023**
Grow policy	Either depthwise or **lossguide**
**Logistic regression**	
Penalty solver	Elasticnet SAGA
C	$[10^{-5}; 1]$
L1 ratio	$[10^{-5}; 1]$

[a]Feature selection rank-orders possible predictors by their chi-squared value. For e.g., the 10-th percentile, the top 10% of features with the highest chi-squared values were kept, the rest were dropped.
[b]Features were constructed for lookbehinds of 30, 180, 365, 730, 1095, 1460 and 1825 days. Which subset to train on was established as a hyperparameter, and one of the above combinations was selected for each training run.

Supplementary Table 4. Descriptive statistics for patients that were eligible for prediction, divided by whether they developed T2D or not.

	Total	T2D	No T2D
**Patients, n**	74880	73520	1360
Female, n (%)	41044 (54.8)	40369 (54.9)	675 (49.6)
Age, median [Q1,Q3]	32.9 [23.0,48.4]	32.6 [22.9,47.8]	53.9 [42.5,68.2]
**Diagnoses, n (%)**			
F0	3552 (4.7)	3425 (4.7)	127 (9.3)
F1	9694 (12.9)	9503 (12.9)	191 (14.0)
F2	7900 (10.6)	7742 (10.5)	158 (11.6)
F3	26004 (34.7)	25551 (34.8)	453 (33.3)
F4	24997 (33.4)	24651 (33.5)	346 (25.4)
F5	3341 (4.5)	3323 (4.5)	18 (1.3)
F6	7925 (10.6)	7836 (10.7)	89 (6.5)
F7	1794 (2.4)	1759 (2.4)	35 (2.6)
F8	4547 (6.1)	4522 (6.2)	25 (1.8)
**Medications, n (%)**			
Top 10 weight gaining antipsychotics	13399 (17.9)	13088 (17.8)	311 (22.9)
Benzodiazepines	13725 (18.3)	13323 (18.1)	402 (29.6)
Benzodiazepine related sleeping agents	11912 (15.9)	11558 (15.7)	354 (26.0)
Clozapine	648 (0.9)	634 (0.9)	14 (1.0)
Lamotrigine	2873 (3.8)	2796 (3.8)	77 (5.7)
Lithium	1467 (2.0)	1431 (1.9)	36 (2.6)
Pregabaline	2425 (3.2)	2351 (3.2)	74 (5.4)
Selected NaSSA	4073 (5.4)	3928 (5.3)	145 (10.7)
SNRI	4931 (6.6)	4784 (6.5)	147 (10.8)
SSRI	9366 (12.5)	9109 (12.4)	257 (18.9)
TCA	2200 (2.9)	2122 (2.9)	78 (5.7)
Valproate	1476 (2.0)	1429 (1.9)	47 (3.5)

Supplementary Table 5. The 100 most important features by information gain (XGBoost with 5 year lookahead)

	Feature Name	Gain
1	730-day mean HbA1c	0.048
**2**	365-day latest weight (kg)	0.042
**3**	730-day max HbA1c	0.033
**4**	365-day latest triglycerides	0.021
**5**	Age (years)	0.019
**6**	365-day max HbA1c	0.019
**7**	730-day max HDL	0.017
**8**	730-day mean urinary_glc	0.017
**9**	365-day min arterial p-Glc	0.017
**10**	365-day min HbA1c	0.017
**11**	730-day mean ALAT	0.016
**12**	365-day min scheduled p-Glc	0.016
**13**	730-day min LDL	0.016
**14**	730-day mean triglycerides	0.016
**15**	730-day latest BMI	0.015
**16**	365-day latest arterial p-Glc	0.015
**17**	730-day min HbA1c	0.015
**18**	365-day latest egfr	0.014
**19**	730-day mean CRP	0.014
**20**	730-day min egfr	0.014
**21**	730-day min arterial p-Glc	0.014
**22**	730-day max unscheduled p-Glc	0.014
**23**	365-day mean HDL	0.013
**24**	730-day max urinary_glc	0.013
**25**	365-day max triglycerides	0.013
**26**	730-day latest albumine creatinine ratio	0.013
**27**	730-day max egfr	0.013
**28**	730-day max albumine creatinine ratio	0.013
**29**	730-day latest triglycerides	0.013
**30**	730-day mean unscheduled p-Glc	0.013
**31**	365-day latest height (cm)	0.013
**32**	365-day mean HbA1c	0.012
**33**	730-day min ALAT	0.012
**34**	730-day max triglycerides	0.012
**35**	365-day max HDL	0.011
**36**	730-day min triglycerides	0.011
**37**	365-day min triglycerides	0.011
**38**	730-day min HDL	0.011
**39**	730-day mean LDL	0.011
**40**	30-day max fasting LDL	0.011
**41**	365-day max unscheduled p-Glc	0.011
**42**	365-day min fasting p-Glc	0.010
**43**	730-day min unscheduled p-Glc	0.010
**44**	365-day max CRP	0.010

**45**	365-day mean unscheduled p-Glc	0.010
**46**	730-day min fasting p-Glc	0.010
**47**	730-day mean HDL	0.009
**48**	365-day max diuretics	0.009
**49**	365-day latest fasting p-Glc	0.009
**50**	365-day mean ALAT	0.009
**51**	730-day max CRP	0.009
**52**	730-day max scheduled p-Glc	0.009
**53**	365-day min ALAT	0.009
**54**	730-day max arterial p-Glc	0.009
**55**	365-day max LDL	0.009
**56**	730-day latest scheduled p-Glc	0.009
**57**	365-day latest HDL	0.008
**58**	730-day max LDL	0.008
**59**	365-day latest ALAT	0.008
**60**	730-day latest F2 disorders	0.008
**61**	365-day latest LDL	0.008
**62**	365-day mean F0 disorders	0.008
**63**	730-day max fasting LDL	0.007
**64**	365-day latest unscheduled p-Glc	0.007
**65**	730-day latest height (cm)	0.007
**66**	365-day min gerd_drugs	0.007
**67**	730-day latest unscheduled p-Glc	0.007
**68**	730-day latest HDL	0.007
**69**	730-day latest LDL	0.007
**70**	730-day latest weight (kg)	0.006
**71**	365-day min HDL	0.006
**72**	730-day latest fasting LDL	0.006
**73**	365-day mean F3 disorders	0.006
**74**	730-day min clozapine	0.006
**75**	365-day max egfr	0.006
**76**	730-day latest HbA1c	0.006
**77**	730-day max F5 disorders	0.005
**78**	730-day max ALAT	0.005
**79**	730-day latest lithium	0.005
**80**	365-day min unscheduled p-Glc	0.005
**81**	730-day latest ALAT	0.005
**82**	365-day min egfr	0.005
**83**	730-day min fasting LDL	0.004
**84**	730-day latest F3 disorders	0.004
**85**	365-day min LDL	0.004
**86**	30-day latest height (cm)	0.004
**87**	365-day min F1 disorders	0.004
**88**	365-day mean statins	0.003
**89**	365-day latest CRP	0.003
**90**	365-day latest top 10 weight gaining antipsychotics	0.003
**91**	730-day max Hyperkinetic disorders	0.001
**92**	730-day latest F7 disorders	0.000

**93**	730-day mean top 10 weight gaining antipsychotics	0.000
**94**	365-day min sleep_apnea	0.000
**95**	30-day latest albumine creatinine ratio	0.000
**96**	30-day min gerd_drugs	0.000
**97**	365-day mean pregabaline	0.000
**98**	30-day min antipsychotics	0.000
**99**	30-day mean sleep_apnea	0.000
**100**	365-day max F6 disorders	0.000

Supplementary Table 6: Performance by predicted positive rate of XGBoost with 1 years of lookahead.

Predicted positive rate	True prevalence	PPV	NPV	Sens	Spec	FPR	FNR	Acc	TP	TN	FP	FN	F1	MCC	% of all events captured	Median years from first positive to T2D
5.0%	0.1%	1.0%	99.9%	45.4%	95.0%	5.0%	54.6%	95.0%	93	179,566	9,370	112	17.9%	19.4%	42.9%	2.5
4.0%	0.1%	1.2%	99.9%	43.9%	96.0%	4.0%	56.1%	96.0%	90	181,460	7,476	115	18.8%	19.4%	38.1%	2.4
3.0%	0.1%	1.4%	99.9%	39.5%	97.0%	3.0%	60.5%	97.0%	81	183,342	5,594	124	20.1%	19.8%	33.3%	2.7
2.0%	0.1%	2.0%	99.9%	36.6%	98.0%	2.0%	63.4%	98.0%	75	185,226	3,710	130	17.6%	16.4%	30.2%	2.4
1.0%	0.1%	3.0%	99.9%	28.3%	99.0%	1.0%	71.7%	98.9%	58	187,089	1,847	147	15.6%	14.8%	22.2%	2.3

Predicted positive rate: The proportion of contacts predicted positive by the model. Since the model outputs a predicted probability, this is a threshold set by us. **True prevalence**: The proportion of contacts that qualified for type 2 diabetes within the lookahead window. **PPV**: Positive predictive value. **NPV**: Negative predictive value. **FPR**: False positive rate. **FNR**: False negative rate. **TP**: True positives. Numbers are service contacts. **TN**: True negatives. Numbers are service contacts. **FP**: False positives. Numbers are service contacts. **FN**: False negatives. Numbers are service contacts. **% of all T2D captured**: Percentage of all patients who developed T2D, who had at least one positive prediction. **Median years from first positive to T2D**: For all patients with at least one true positive, the number of days from their first positive prediction to being labelled as T2D.

Supplementary Table 7: Performance by predicted positive rate of XGBoost with 2 years of lookahead.

Predicted positive rate	True prevalence	PPV	NPV	Sens	Spec	FPR	FNR	Acc	TP	TN	FP	FN	F1	MCC	% of all events captured	Median years from first positive to T2D
5.0%	0.3%	3.6%	99.8%	52.6%	95.2%	4.8%	47.4%	95.0%	251	132,260	6,722	226	7.9%	13.8%	43.0%	2.4
4.0%	0.3%	4.4%	99.8%	51.8%	96.2%	3.8%	48.2%	96.0%	247	133,641	5,341	230	8.5%	14.2%	41.9%	2.4
3.0%	0.3%	5.1%	99.8%	44.4%	97.1%	2.9%	55.6%	97.0%	212	135,008	3,974	265	9.3%	14.2%	33.7%	2.3
2.0%	0.3%	6.7%	99.8%	39.6%	98.1%	1.9%	60.4%	97.9%	189	136,357	2,625	288	10.6%	15.6%	30.2%	2.1
1.0%	0.3%	6.5%	99.7%	19.1%	99.1%	0.9%	80.9%	98.8%	91	137,675	1,307	386	12.4%	10.6%	18.6%	2.1

Predicted positive rate: The proportion of contacts predicted positive by the model. Since the model outputs a predicted probability, this is a threshold set by us. **True prevalence**: The proportion of contacts that qualified for type 2 diabetes within the lookahead window. **PPV**: Positive predictive value. **NPV**: Negative predictive value. **FPR**: False positive rate. **FNR**: False negative rate. **TP**: True positives. Numbers are service contacts. **TN**: True negatives. Numbers are service contacts. **FP**: False positives. Numbers are service contacts. **FN**: False negatives. Numbers are service contacts. **% of all T2D captured**: Percentage of all patients who developed T2D, who had at least one positive prediction. **Median years from first positive to T2D**: For all patients with at least one true positive, the number of days from their first positive prediction to being labelled as T2D.

Supplementary Table 8: Performance by predicted positive rate of XGBoost with 3 years of lookahead.

Predicted positive rate	True prevalence	PPV	NPV	Sens	Spec	FPR	FNR	Acc	TP	TN	FP	FN	F1	MCC	% of all events captured	Median years from first positive to T2D
5.0%	0.7%	5.7%	99.6%	43.4%	95.3%	4.7%	56.6%	94.9%	401	133,374	6,648	522	11.8%	14.3%	37.9%	2.9
4.0%	0.7%	6.7%	99.6%	41.0%	96.2%	3.8%	59.0%	95.9%	378	134,760	5,262	545	13.0%	6.0%	34.8%	2.9
3.0%	0.7%	8.0%	99.6%	36.8%	97.2%	2.8%	63.2%	96.8%	340	136,128	3,894	583	14.4%	16.5%	31.8%	2.7
2.0%	0.7%	9.3%	99.5%	28.3%	98.2%	1.8%	71.7%	97.7%	261	137,464	2,558	662	15.2%	15.2%	25.8%	2.7
1.0%	0.7%	10.0%	99.4%	15.3%	99.1%	0.9%	84.7%	98.5%	141	138,752	1,270	782	14.7%	28.2%	13.6%	2.4

Predicted positive rate: The proportion of contacts predicted positive by the model. Since the model outputs a predicted probability, this is a threshold set by us. **True prevalence**: The proportion of contacts that qualified for type 2 diabetes within the lookahead window. **PPV**: Positive predictive value. **NPV**: Negative predictive value. **FPR**: False positive rate. **FNR**: False negative rate. **TP**: True positives. Numbers are service contacts. **TN**: True negatives. Numbers are service contacts. **FP**: False positives. Numbers are service contacts. **FN**: False negatives. Numbers are service contacts. **% of all T2D captured**: Percentage of all patients who developed T2D, who had at least one positive prediction. **Median years from first positive to T2D**: For all patients with at least one true positive, the number of days from their first positive prediction to being labelled as T2D.

Supplementary Table 9: Performance by predicted positive rate of XGBoost with 4 years of lookahead.

Predicted positive rate	True prevalence	PPV	NPV	Sens	Spec	FPR	FNR	Acc	TP	TN	FP	FN	F1	MCC	% of all events captured	Median years from first positive to T2D
5.0%	1.1%	10.6%	99.4%	48.5%	95.5%	4.5%	51.5%	95.0%	450	80,458	3,810	478	13.7%	21.2%	43.0%	3
4.0%	1.1%	12.4%	99.4%	45.7%	96.5%	3.5%	54.3%	95.9%	424	81,283	2,985	504	14.8%	22.3%	39.3%	3
3.0%	1.1%	14.4%	99.3%	39.8%	97.4%	2.6%	60.2%	96.8%	369	82,080	2,188	559	15.5%	22.6%	32.7%	2.7
2.0%	1.1%	11.5%	99.1%	21.1%	98.2%	1.8%	78.9%	97.4%	196	82,759	1,509	732	16.0%	14.3%	21.5%	2.7
1.0%	1.1%	15.0%	99.1%	13.8%	99.1%	0.9%	86.2%	98.2%	128	83,544	724	800	13.5%	13.5%	15.0%	1.9

Predicted positive rate: The proportion of contacts predicted positive by the model. Since the model outputs a predicted probability, this is a threshold set by us. **True prevalence**: The proportion of contacts that qualified for type 2 diabetes within the lookahead window. **PPV**: Positive predictive value. **NPV**: Negative predictive value. **FPR**: False positive rate. **FNR**: False negative rate. **TP**: True positives. **TN**: True negatives. **FP**: False positives. **FN**: False negatives. **% of all T2D captured**: Percentage of all patients who developed T2D, who had at least one positive prediction. **Median years from first positive to T2D**: For all patients with at least one true positive, the number of days from their first positive prediction to being labelled as T2D.

Supplementary Table 10: Performance by predicted positive rate of XGBoost with only age, sex and HbA1c with 4 years of lookahead

Predicted positive rate	True prevalence	PPV	NPV	Sens	Spec	FPR	FNR	Acc	TP	TN	FP	FN	F1	MCC	% of all T2D captured	Median years from first positive to T2D
5.0%	1.1%	11.6%	99.1%	39.9%	95.5%	4.5%	60.1%	94.7%	1,899	309,125	14,522	2,866	17.9%	19.4%	37.9%	2.6
4.0%	1.1%	12.8%	99.0%	35.3%	96.5%	3.5%	64.7%	95.6%	1,684	312,193	11,454	3,081	18.8%	19.4%	33.4%	2.6
3.0%	1.1%	14.9%	99.0%	30.8%	97.4%	2.6%	69.2%	96.4%	1,467	315,256	8,391	3,298	20.1%	19.8%	28.3%	2.6
2.0%	1.1%	15.2%	98.8%	21.0%	98.3%	1.7%	79.0%	97.2%	999	318,077	5,570	3,766	17.6%	16.4%	20.1%	2.6
1.0%	1.1%	19.1%	98.7%	13.2%	99.2%	0.8%	86.8%	97.9%	628	320,987	2,660	4,137	15.6%	14.8%	13.8%	2.4

Predicted positive rate: The proportion of contacts predicted positive by the model. Since the model outputs a predicted probability, this is a threshold set by us. **True prevalence**: The proportion of contacts that qualified for type 2 diabetes within the lookahead window. **PPV**: Positive predictive value. **NPV**: Negative predictive value. **FPR**: False positive rate. **FNR**: False negative rate. **TP**: True positives. **TN**: True negatives. **FP**: False positives. **FN**: False negatives. **% of all T2D captured**: Percentage of all patients who developed T2D, who had at least one positive prediction. **Median years from first positive to T2D**: For all patients with at least one true positive, the number of days from their first positive prediction to being labelled as T2D.

Supplementary Discussion

Selection of wash-in periods

Since Danish guidelines recommend measuring HbA1c at least every 6 months for patients with T2D,[6] it can be argued that a 6-month wash-in should eliminate all prevalent cases. However, Supplementary Figure 3 shows that a 2-year wash-in was required for T2D incidence to stabilise. Similarly, for patients moving to the Central Denmark Region, we enforced a 2-year wash-in period in which no predictions were issued. If they met the criteria for T2D in this period, we assumed that they had been diagnosed outside of the Central Denmark Region and did not issue any further predictions.

Validity of the definition of T2D

Defining T2D in cohort studies comes with challenges, since T2D models may misclassify type 1 diabetes as T2D due to similar diagnostic criteria.[7] To address this, we excluded contacts with a prior type 1 diabetes diagnosis or insulin use, and only made predictions for patients aged ≥18 years, which should largely solve this issue.

Effects of potential model implementation

When prediction models are implemented, they should affect behaviour, for example by decreasing delay to diagnostic testing. Specifically, implementing a T2D prediction model would likely induce more relevant HbA1c-measurements. These model-induced measurements should improve the next prediction of the model, meaning that predictions following a positive prediction are likely less accurate in our dataset than they would be following implementation.
